# A multi-step transcriptional cascade underlies vascular regeneration ***in vivo***

**DOI:** 10.1038/s41598-018-23653-3

**Published:** 2018-04-03

**Authors:** Aditya S. Shirali, Milagros C. Romay, Austin I. McDonald, Trent Su, Michelle E. Steel, M. Luisa Iruela-Arispe

**Affiliations:** 1 Department of Surgery, University of California at Los Angeles, Los Angeles, CA 90095 USA; 2 Department of Molecular, Cell, and Developmental Biology, University of California at Los Angeles, Los Angeles, CA 90095 USA; 3 Molecular Biology Institute, University of California at Los Angeles, Los Angeles, CA 90095 USA; 4 Department of Biological Chemistry, David Geffen School of Medicine, University of California at Los Angeles, Los Angeles, CA 90095 USA; 5 Institute for Quantitative and Computational Biology, University of California, Los Angeles, Los Angeles, CA 90095 USA

## Abstract

The molecular mechanisms underlying vascular regeneration and repair are largely unknown. To gain insight into this process, we developed a method of intima denudation, characterized the progression of endothelial healing, and performed transcriptome analysis over time. Next-generation RNA sequencing (RNAseq) provided a quantitative and unbiased gene expression profile during *in vivo* regeneration following denudation injury. Our data indicate that shortly after injury, cells immediately adjacent to the wound mount a robust and rapid response with upregulation of genes like *Jun*, *Fos*, *Myc*, as well as cell adhesion genes. This was quickly followed by a wave of proliferative genes. After completion of endothelial healing a vigorous array of extracellular matrix transcripts were upregulated. Gene ontology enrichment and protein network analysis were used to identify transcriptional profiles over time. Further data mining revealed four distinct stages of regeneration: shock, proliferation, acclimation, and maturation. The transcriptional signature of those stages provides insight into the regenerative machinery responsible for arterial repair under normal physiologic conditions.

## Introduction

Our current understanding of endothelial growth is largely rooted in studies that focused on either developmental or pathological angiogenesis^1,2^. In adult vessels, vascular expansion is usually stimulated by an insult that results in either cytokine production or hypoxia^3^. These events quickly trigger a complex interplay of downstream signaling pathways that affect junctional dynamics, induce vasodilation, and promote vascular permeability^4,5^. Disruption of the basement membrane with the subsequent departure of endothelial cells from pre-existing vessels initiates the formation of a sprout that expands through endothelial cell proliferation and migration^6–8^. The final phase entails the formation of a vascular lumen, the acquisition of a basement membrane and investment of mural cells^9^. All of these stages strictly depend on a wide array of cytokines, tyrosine kinases, Notch signaling proteins, junctional proteins, and integrins that orchestrate complex and sequential changes in the endothelium that entail: release from homeostasis, migration, proliferation, and return to homeostasis^8,10^. After morphogenesis is complete, endothelial survival and/or apoptosis relies on the provision of nutrients and shear stress signals via blood flow states, with high flow states inducing endothelial survival and low flow states inducing apoptosis^11,12^. The network of newly developed arteries subsequently undergoes vascular remodeling or pruning into a hierarchical network of vessels^13^. Nonetheless, injury of large vessels differs from angiogenesis, as regeneration of the tunica intima must occur in the absence of vascular sprouting and in continuity with the endothelial monolayer. In this context, vascular regeneration following *in situ* injury within large- or medium-sized arteries, such as following traumatic injury or surgical intervention, remains relatively unknown.

Previous studies have shown that injury to large arteries, such as the carotid and coronary arteries, induced a program of regeneration that results in vascular repair^14–16^. Rabbit animal models were used extensively to understand the biological responses to injury induced by deployment of stents or from hypercholesterolemia^17–19^. Those studies, however, were primarily focused on smooth muscle growth related to restenosis and neointimal hyperplasia with little focus on the endothelium^20–23^. Furthermore, the findings from the endothelium were confounded by the lack of information on proliferation and the limited visibility offered by cross-sections of the endothelial layer. Molecular regenerative information in these models has also been hindered by the limited material isolated from the carotid or femoral arteries, the inability to obtain a reproducible injury, and the difficulty of producing an area of denudation completely devoid of endothelium. These factors have stalled flow of information that have been relatively easy to obtain in other tissues^24–29^.

As such, we sought to create a new model of arterial denudation injury to allow for gene expression profiling and evaluate the transcriptional signatures associated with vascular regeneration following mechanical arterial injury in the context of a fully functional vessel. This approach was combined with flushing RNA lysis buffer directly in the lumen of the aorta, similar to what has been previously done to study the effects of flow disturbances in the carotid, to obtain intima-enriched aortic RNA of regenerating vessels^30,31^. In the process, it became clear that vascular regeneration follows four clearly distinct stages of regeneration that, with the exception of proliferation, have little overlap with the process of vascular expansion known as angiogenesis.

## Results

### Healing of arterial denudation injury is marked by proliferation that promotes wound closure

**Figure 1:**
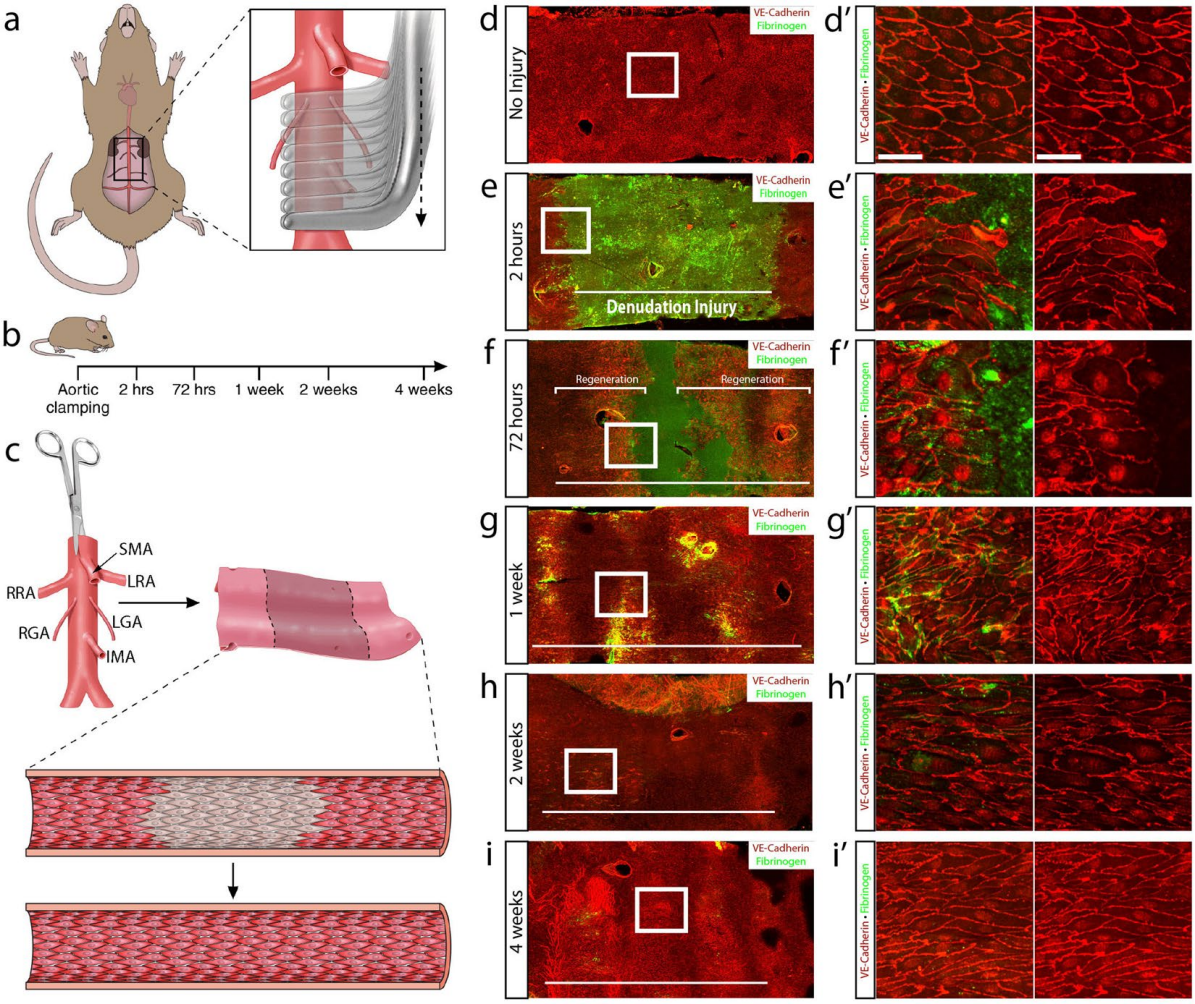
Sequential aortic cross clamping produces aortic arterial denudation injury. (**a**) Schematic representation of the aortic clamping procedure in mice. Sequential clamping of the infrarenal abdominal aorta from below the renal arteries to the iliac bifurcation. (**b**) Aortas were subsequently harvested at 2 hours (n = 6), 72 hours (n = 6), 1 week (n = 5), 2 weeks (n = 4) and 4 weeks (n = 4) following denudation injury. (**c**) Aortas were longitudinally transected and immunohistochemistry (IHC) was performed *en face*. (**d**) VE-cadherin (red) identifying the endothelial monolayer and fibrinogen (green) IHC of a non-injured aorta. (**e**) Aortic cross clamping produced a 1575 μm injury length of denuded endothelium, marked by a horizontal white line, at 2 hours following injury. (**f**–**i**) Fibrinogen IHC showed the denudation area and VE-cadherin identified the endothelial monolayer at 72 hours (f), 1 week (g), 2 weeks (h) and 4 weeks(i). The white horizontal bar marks the original denudation injury length. (d’–i’) Higher magnification of the boxed areas. Scale bar: 40 μm.

Cross clamping of the mouse infrarenal abdominal aorta in a sequential fashion was used to generate a reproducible endothelial denudation model (Fig. 1a). The imposed injury extended from below the renal arteries to the iliac bifurcation resulting in an injury of approximately 1700 to 2400 μm in length and corresponded to 15–20% of the mouse infrarenal abdominal aorta (Suppl. Fig. 1a,b). We then allowed for progressive repair of the wound by closing the mouse and evaluating the status of regeneration at 2 hours, 72 hours, 1 week, 2 weeks and 4 weeks following denudation injury (Fig. 1b), transected the aorta longitudinally (Fig. 1c) and performed *en face* immunohistochemistry (Fig. 1d–i). VE-cadherin and fibrinogen were used to identify endothelial cell junctions and denudation injury, respectively. Immunohistochemistry confirmed that the procedure produced a contiguous area devoid of endothelium and of the predicted length 2 hours after injury (Fig. 1e and e’). Interestingly, the injury did not remove the basement membrane, as per evaluation of type IV Collagen (Suppl. Fig. 1c). At 72 hours, the endothelial wound area was significantly reduced due to regeneration of the endothelial monolayer at both the proximal and distal sites of injury. Importantly, the process of endothelial repair was equivalent upstream and downstream of flow. Regenerating endothelial cells at 72 hours were marked by hypertrophy, elongation, and decreased VE-cadherin along the apical periphery of the leading edge of cells (Fig. 1f and f’). Upon wound closure at 1 week, immunohistochemistry identified large and disorganized clusters of cells that were denser in number, smaller in diameter, and not fully oriented in the direction of blood flow (Fig. 1g and g’). The reorganization of endothelial cells persisted at 2 weeks (Fig. 1h and h’) until finally at 4 weeks a completely closed monolayer of endothelial cells oriented in the direction of blood flow was observed (Fig. 1i–i’).

**Figure 2:**
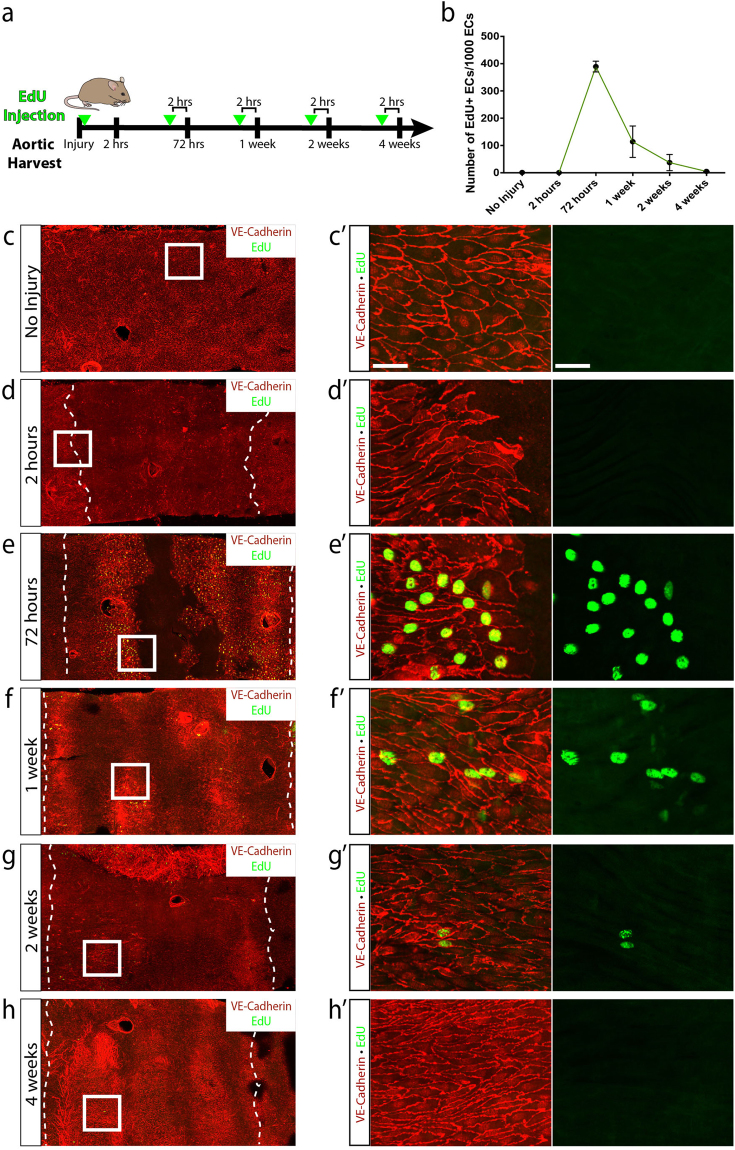
Regeneration of the endothelial monolayer is marked by proliferation that initiates rapidly after injury and promotes wound closure. (**a**) Schematic representation of the experimental design. Mice were injected with EdU 2 hours prior to harvesting of aortas. (**b**) Number of EdU-positive endothelial cells per 1,000 endothelial cells (mean ± SEM) without injury (n = 6) and 2 hours (n = 6), 72 hours (n = 6), 1 week (n = 5), 2 weeks (n = 4) and 4 weeks (n = 4) following injury. (**c**–**h**) EdU-positive endothelial cells following injury at the indicated time points (green). VE-cadherin was used to visualize endothelial junctions (red). (c’–h’) Higher magnification IHC of the boxed areas showed EdU-positive endothelial cells at 72 hours, 1 week and 4 weeks following injury. Scale bar: 40 μm.

We next investigated the contribution of endothelial cell proliferation to healing of arterial denudation injury. Mice were injected with EdU 2 hours prior to harvesting of aortas to identify cells that had entered the cell cycle (S phase) and were actively proliferating (Fig. 2a). Quantification of EdU-positive cells within the area of denudation during each time point of regeneration further validated these observations. Compared to the non-injured aorta with 1.14 ± 0.595 EdU-positive endothelial cells per 1000 endothelial cells (mean ± SEM), the subsequent time points showed 0.554 ± 0.355, 389 ± 19.8, 114 ± 57.6, 37.4 ± 29.8, and 5.05 ± 0.707 at 2 hours, 72 hours, 1 week, 2 weeks and 4 weeks, respectively (Fig. 2b). No EdU-positive cells were found at 2 hours following injury proximally or distally to the wound (Fig. 2d and d’). By 72 hours post-injury a significant increase in proliferating endothelial cells was noted in areas restricted to the wound margins (Fig. 2e and e’). Interestingly, endothelial cells continued to demonstrate EdU-positivity at 1 week and 2 weeks following injury (Fig. 2f–g’) well after the injury was completely repaired, suggesting that wound closure was insufficient to cease proliferation. Finally, aortas harvested 4 weeks following injury demonstrated a minimal number of EdU-positive cells and were similar to the non-injured aorta (Fig. 2h and h’). These data showed no enhancement of proliferation 2 hours following injury, marked cell-cycle entry at 72 hours contributing to wound closure, and persistence of endothelial cell proliferation at 1 week and 2 weeks.

### Identification of transcriptional signatures associated with vascular regeneration

To elucidate the molecular underpinnings that trigger and drive regeneration of the tunica intima, we sought to obtain and analyze transcriptomes for each of the time points following arterial denudation. However, we first sought to determine whether a previously developed method for obtaining intima-enriched RNA may be applied along the area of denudation to produce transcriptional readouts specific to the intimal layer and avoid dilution effects from the large number of smooth muscle and adventitial cells^31^. As such, intima-enriched aortic RNA was isolated and compared to whole aortic RNA by RNAseq (Suppl. Fig. S2a). Evaluation of the aorta subjected to flushing of RNA lysis buffer demonstrated absence of endothelial cells as per ERG immunocytochemistry, suggesting enrichment of endothelial cells in the lysis buffer with minimal removal of smooth muscle cells, as per alpha-smooth muscle actin (Suppl. Fig. S2b,b’). Principal component analysis (PCA) showed clustering of the intima-enriched aortic RNA transcripts and clear distinction from the cohort of whole-aortic RNA samples, as well as the flushed aorta samples (smooth muscle cell-enriched) (Suppl. Fig. S2c). Unbiassed hierarchical clustering also revealed low variance within the intima-enriched samples and segregation from whole-aorta and tunica media-enriched (flushed aorta) samples (Suppl. Fig. S2d). Examination of the relative abundance of transcripts from the endothelium, smooth muscle and inflammatory cells showed a paucity of smooth muscle-specific transcripts, *Notch 3* and *Calponin*, and enhanced endothelial cell-specific transcripts, *VE-cadherin*, *PECAM-1*, *vWF and VCAM-1*, in the intima-enriched aortic RNA compared to whole aortic RNA (Suppl. Fig. S2e). Taken together, we found this method of RNA extraction produced a transcriptome reflective of intimal and endothelial cell-enrichment compared to homogenized whole aortic RNA.

**Figure 3:**
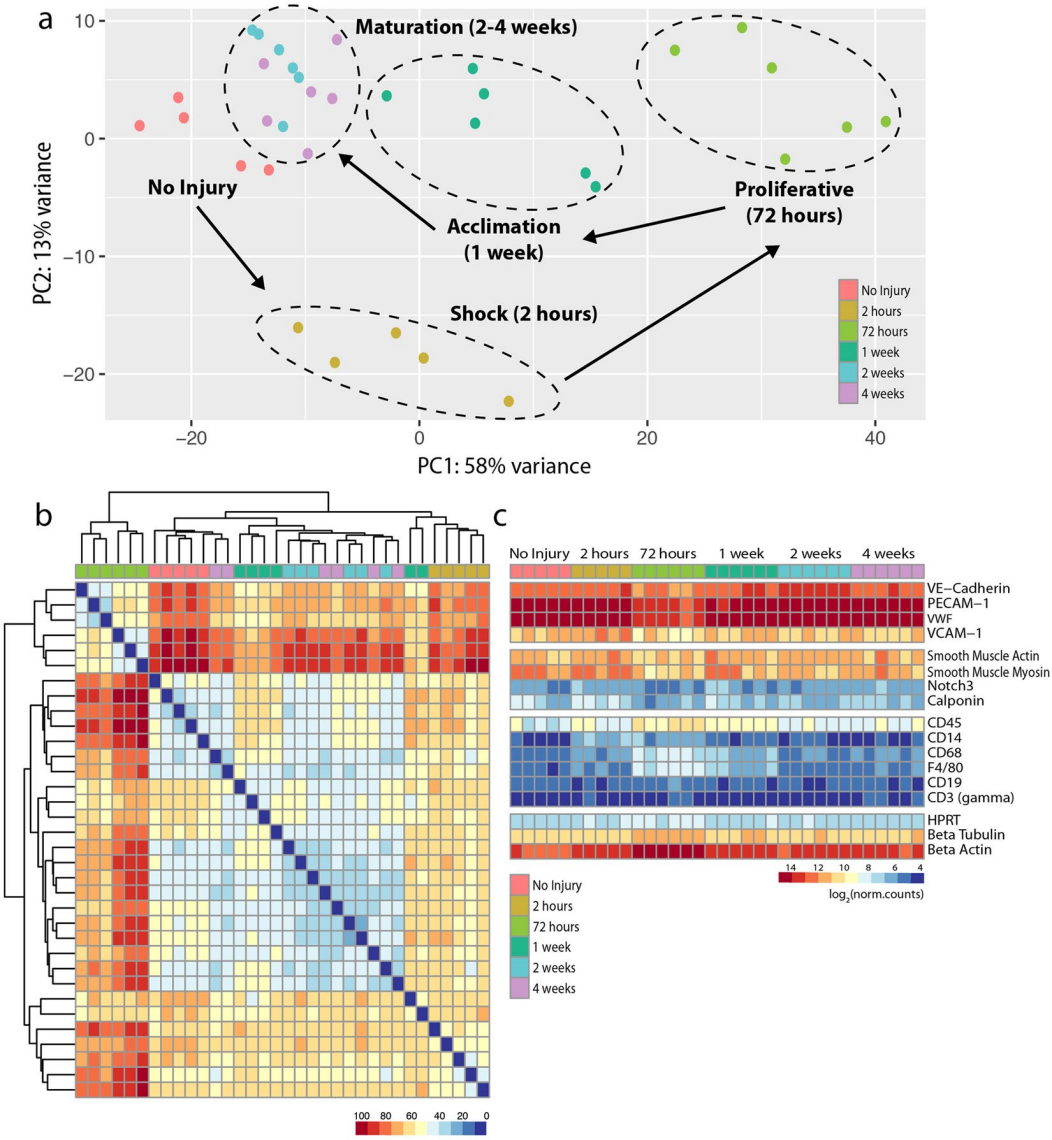
Transcriptomic analysis identifies unique transcriptional signatures associated with the progression of vascular regeneration. (**a**) Principal Component Analysis (PCA) demonstrated the source of variance in our data and identified unique stages of wound healing: shock at 2 hours (n = 5), proliferative at 72 hours (n = 6), acclimation at 1 week (n = 6) and maturation at 2 and 4 weeks (n = 6 at both time points), with convergence toward the non-injured aortic transcripts (n = 5). (**b**) Heat map showing the Euclidean distances between the samples as calculated from the regularized log transformation. (**c**) Heat map showing relative abundance of transcripts from endothelial, smooth muscle, inflammatory, and house-keeping genes. Note enrichment in endothelial-specific markers at all time points.

RNAseq was then performed for the non-injured aorta and at each time point following denudation injury. Comparing the differentially expressed genes (DEGs) from denuded aortas to the non-injured aortas, we observed 359 DEGs at 2 hours, 2046 DEGs at 72 hours, 526 DEGs at 1 week, 180 DEGs at 2 weeks, and 104 DEGs at 4 weeks using an adjusted p-value of 2.6898 × 10^−6^. PCA demonstrated close clustering of individual samples from each time point after injury as well as separation between different time points, validating the low variance within samples and high variance between no injury and the regeneration time points of 2 hours, 72 hours and 1 week (Fig. 3a). Interestingly, the 2 week and 4 week time points showed low variance, as confirmed by the distance heat map in which samples from both the 2 week and 4 week repair groups were intermixed (Fig. 3b). As such, these time points were combined for subsequent analysis. In fact, the distribution of samples by PCA allowed us to identify four unique transcriptional signatures associated with the progression of vascular regeneration. Interestingly, these transcriptional signatures gain proximity over time towards the non-injured endothelium, as indicated by the arrows, indicating progressive return to homeostasis.

Sub-analysis of the relative abundance of transcripts for endothelial, smooth muscle and inflammatory cells showed an enrichment of endothelial-specific transcripts, *VE-cadherin* and *PECAM-1*, with average read numbers 10,538 ± 723 (mean ± SEM) and 33,976 ± 1,416, respectively, as well as a relative low abundance of smooth muscle-specific transcripts, *Notch3* and *Calponin*, with average read numbers 104 ± 12.8 and 177 ± 20.3, respectively (Fig. 3c, Supplementary Data S1). Interestingly, an increase in inflammatory cell transcripts, *CD45*, *CD68*, and *F4/80*, was noted during the 72 hour and 1 week time points suggesting presence of inflammatory cells in the tunica intima during wound closure that contributed to the transcriptional read outs of the intima-enriched aortic RNA (Fig. 3c). This was confirmed by immunohistochemistry of CD45 that showed adherence of inflammatory cells in the regenerating endothelium at 72 hours, 1 week, 2 weeks and 4 weeks following injury (Suppl. Fig. S3). CD45-positive cells were observed clustering along the proximal edge of proliferating endothelium at 72 hours and disorganized endothelium at 1 week, and were subsequently found adhering to endothelium. Thus, the transcriptional signatures obtained for each stage of vascular regeneration were a reflection of the regenerating intima, composed of regenerating endothelium and a few adherent CD45-positive cells.

### Rapid and robust responses to injury in the endothelial transcriptome

**Figure 4:**
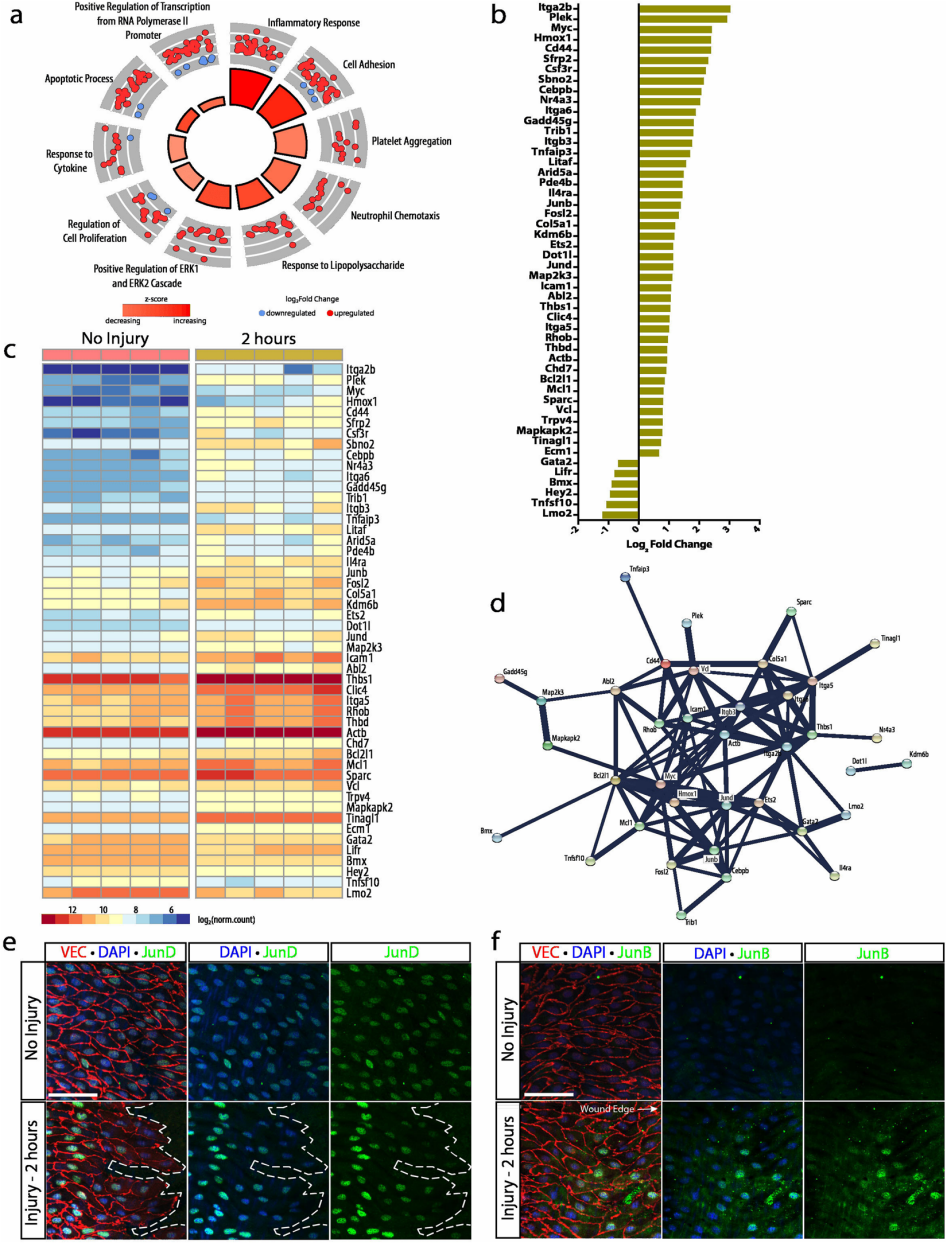
Initial injury is characterized by cell stress, transcriptional regulation and cell adhesion. (**a**) Gene ontology enrichment identified inflammatory response and cell adhesion as significantly upregulated processes, among others, 2 hours after injury. (**b**) The top 50 differentially expressed genes (identified by lowest p-value) organized by fold change. (**c**) Heat map showing differences in normalized counts of the top 50 differentially expressed genes between no injury (n = 5) and 2 hours (n = 5). (**d**) Protein-protein interaction network of top 50 differentially expressed genes via STRING analysis identified clusters specific to cell stress and cell adhesion. (**e**) JunD expression 2 hours following injury (green) as revealed by immunohistochemistry. VE-cadherin and DAPI denote endothelial junctions (red) and nuclei (blue), respectively. Scale bar: 60 μm (**f**) Immunohistochemistry for JunB expression 2 hours following injury (green). VE-cadherin and DAPI reveal endothelial junctions (red) and nuclei (blue), respectively. Scale bar: 60 μm.

Differential gene expression of the non-injured aorta versus 2 hours following injury identified 359 DEGs, of which 313 genes were upregulated and 46 genes were downregulated. Gene ontology enrichment of this cohort of DEGs highlighted 213 biological processes (*p* < 0.05, Supplementary Data S2). After filtering for redundant genes, the top 10 GOs were identified and presented in Fig. 4a; these included 143 distinct genes, many of which were redundant to multiple biological processes. The 143 genes were further filtered by mean read count greater than 200 and lowest p-value to identify the top 50 DEGs. Positive regulation of transcription from RNA Polymerase II promoter, cell adhesion, and apoptotic process were identified to be the most prominent activities represented among the top 50 DEGs with 17, 14, and 11 DEGs, respectively. Gene ontology enrichment suggested a shift toward cell stress, cell adhesion, and transcriptional regulation at 2 hours following injury.

Genes associated with apoptosis and cell stress with a greater than 2-fold increase included *Hmox1*, *Sfrp2*, *Gadd45g*, *Tnfaip3*, *Litaf*, and *Clic4*. A similar increase was also noted in *Myc*, *Sbno2*, *Cebpb*, *Nr4a3*, *Itga6*, *Arid5a*, *Junb*, *Fosl2*, *Kdm6b*, *Ets2*, *Dot1l*, and *Abl2* within the positive regulation of RNA Polymerase II promoter process (Fig. 4b and c). Interestingly, genes associated with apoptosis and transcriptional regulation encoded for proteins that clustered together when analyzed for protein-protein interactions via STRING networking analysis and showed significant interactions between BCL2L1, Myc, Mcl-1, JunB, FOSL2, JunD, Hmox1 and Ets2 with confidence scores greater than 0.9 (Fig. 4d). On the other hand, genes associated with cell adhesion with a greater than 2-fold increase included *Itga2b*, *Cd44*, *Csf3r*, *Itga6*, *Itgb3*, *Col5a1*, *Icam1*, *Abl2*, *Thbs1*, and *Itga5*. These genes encoded for proteins that clustered separately from the knot characterized by cell stress and had protein-protein interaction confidence scores greater than 0.9 with the exception of collagen α-1(V) chain and G-CSF receptor, encoded by *Csf3r* (Supplementary Data S2). Protein-protein interaction networking identified two principal clusters from the top 50 DEGs identified, one which functioned to influence transcriptional regulation during cell stress and the other associated with cell adhesion. Validation of the RNAseq analysis was performed by immunocytochemistry of a cohort of genes. Shown are expression of JunD and JunB. Interestingly the findings indicate that the source of the upregulation in these genes originate from cells immediately adjacent to the injury (Fig. 4e,f). These results illustrated that the first stage of vascular regeneration is characterized by a rapid response (as early as 2 hours) from the cohort of cells right next to the removed endothelium. The response is characterized by shock with a transcriptome of cell stress, transcriptional activation, and cell adhesion.

### Pronounced increases in cell cycle transcripts characterizes the proliferative stage

**Figure 5:**
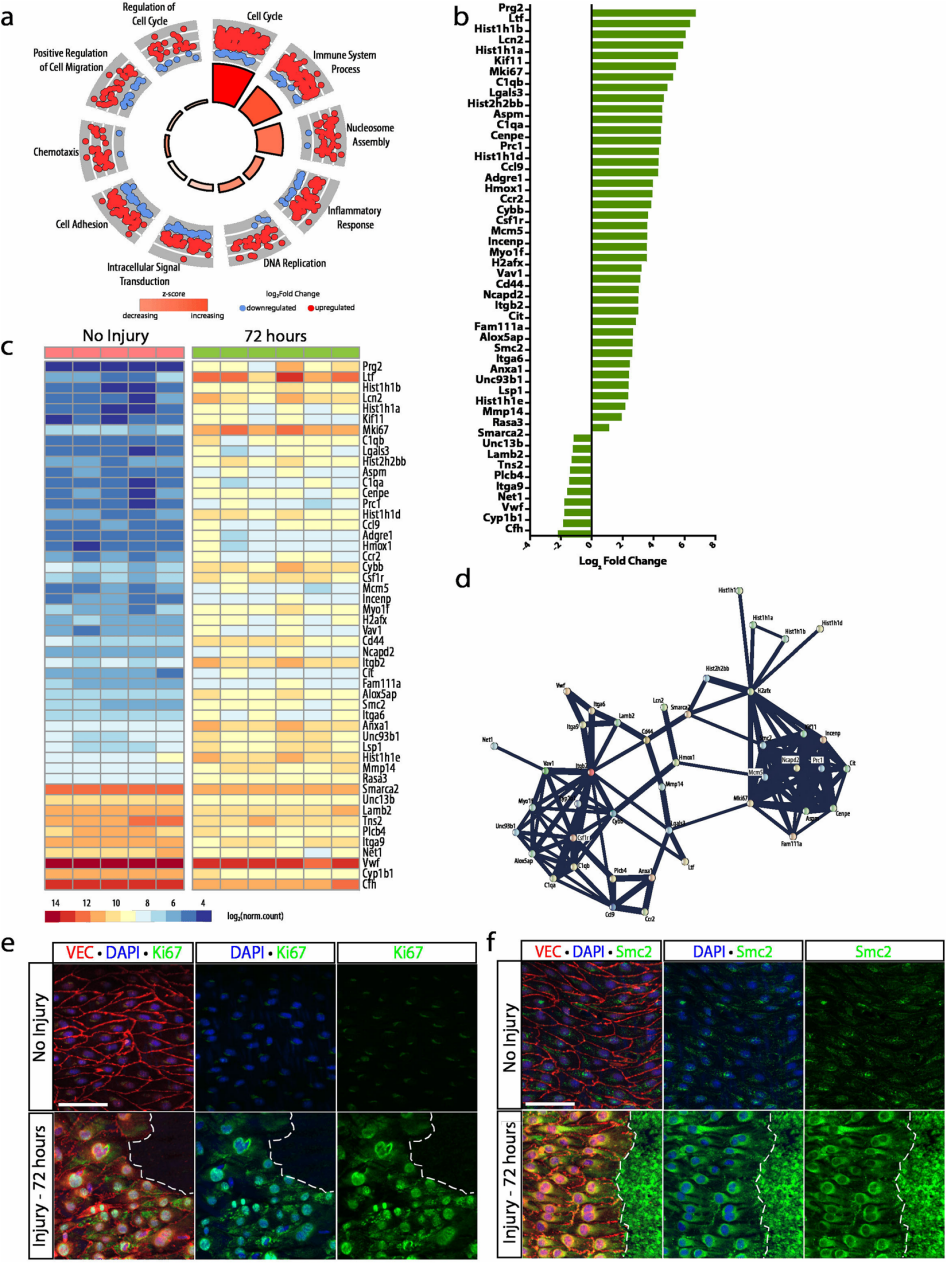
Marked proliferation is characterized by enhancement in cell cycle transcripts. (**a**) Gene ontology enrichment identified cell cycle, immune system process, and nucleosome assembly as the most significantly upregulated processes 72 hours after injury. (**b**) The top 50 differentially expressed genes (identified by lowest p-value) organized by fold change. (**c**) Heat map showing differences in normalized counts of the top 50 differentially expressed genes between no injury (n = 5) and 72 hours (n = 6). (**d**) Protein-protein interaction network of top 50 differentially expressed genes via STRING analysis identified protein clusters specific to proliferation and inflammation. (**e**) Ki67 expression 72 hours following injury (green) by immunohistochemistry. VE-cadherin and DAPI denote endothelial junctions (red) and nuclei (blue), respectively. Scale bar: 60 μm (**f**) Immunohistochemistry reveals Smc2 expression 72 hours following injury (green). VE-cadherin and DAPI reveal endothelial junctions (red) and nuclei (blue), respectively. Scale bar: 60 μm.

The proliferative stage exhibited 2046 DEGs between the non-injured and 72 hour injury time point, a marked increase in differential expression compared to other time points. Of the 2046 DEGs, 1310 genes were upregulated and 736 genes were downregulated. Gene ontology enrichment of this cohort of genes identified 412 biological process (*p < *0.05, Supplementary Data S3). The ten most significant GOs were selected and included: cell cycle, immune system process, nucleosome assembly, inflammatory response, DNA replication, intracellular signal transduction, cell adhesion, chemotaxis, positive regulation of cell migration, and regulation of cell cycle (Fig. 5a). These 10 biological activities incorporated 584 of the 2046 DEGs, and were further filtered by read count and p-value to select the top 50 DEGs. Immune system process and cell cycle were the two most prominent activities represented by the top 50 DEGs with 12 and 11 DEGs respectively.

Cell cycle genes exhibiting a greater than 2-fold increase included: *Kif11*, *Mki67*, *Aspm*, *Cenpe*, *Prc1*, *Mcm5*, *Incenp*, *H2afx*, *Ncapd2*, *Cit*, and *Smc2* (Fig. 5b). These genes demonstrated pronounced increases in gene expression, from 6-fold to 43-fold. Average read counts for *Smc2*, the gene with the lowest fold change in this cohort, changed from 98.0 ± 12.9 (mean ± SEM) in non-injured aortas to 420.4 ± 65.0 at the 72 hour time point. Whereas average read counts for *Kif11*, the gene with the highest fold change in this cohort, increased from 13.2 ± 3.35 in non-injured aortas to 426 ± 49.3 at the 72 hour time point (Fig. 5c). Analysis of read counts demonstrated the extremely low expression profile of these genes in non-injured aortas and the pronounced increase in cell cycle transcripts found 72 hours after injury consistent with the increase in proliferation documented by EdU incorporation at this time point. All proteins encoded by these genes formed a very tight cluster via protein-protein interaction network analysis with confidence scores greater than 0.9 (Fig. 5d, Supplementary Data S3). This finding, unsurprisingly, correlated with the presence of the mitotically-active cells along the wound margin of the regenerating endothelial monolayer. In agreement with an enhancement of CD45-positive cells observed in immunohistochemistry (Suppl. Fig. S3), genes relating to the immune system process had a prominent enrichment at 72 hours. Immune system process genes exhibiting a greater than 2-fold change included *Prg2*, *Ltf*, *Lcn2*, *C1qb*, *Lgals3*, *C1qa*, *Ccl9*, *Adgre1*, *Csf1r*, *Anxa1*, *Unc93b1*, and *Cfh* (Fig. 5b and c). Similar to cell cycle genes, these genes demonstrated pronounced fold changes in expression levels. However, proteins encoded by these genes showed loose clustering in protein-protein interaction network analysis with confidence scores ranging from 0.44 to 0.85 with the exception of the C1qA-C1qB interaction which demonstrated a 0.99 confidence score (Supplementary Data S3) (Fig. 5d). Validation of the transcriptional data is shown for Ki67 and Smc2. Both proteins are poorly expressed in the area of the aorta far from the injury and drastically increased in cells adjacent to the wound area (Fig. 5e,f).

### Transcripts for extracellular matrix and inflammatory cell adherence characterize the acclimation stage of wound repair

**Figure 6:**
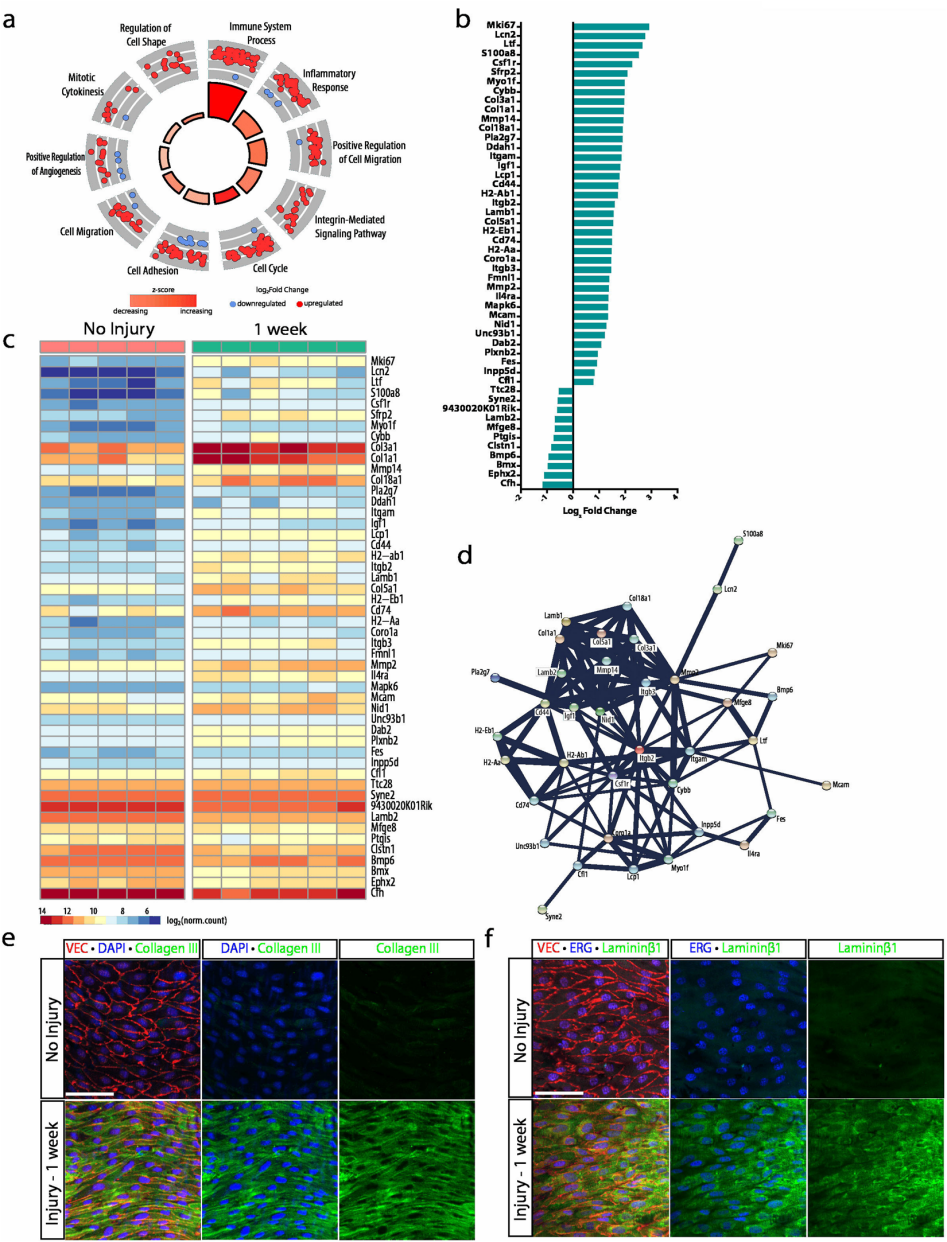
Acclimation is characterized by enhancement of cell adhesion and inflammatory transcripts. (**a**) Gene ontology enrichment identified immune system process, positive cell migration and cell adhesion among the top ten most significant biological processes at 1 week following injury. (**b**) The top 50 differentially expressed genes (identified by lowest p-value) organized by fold change. (**c**) Heat map showing differences in normalized counts of the top 50 differentially expressed genes between no injury (n = 5) and 1 week (n = 6). (**d**) Protein-protein interaction network of top 50 differentially expressed genes via STRING analysis identified a cluster specific to basement membrane and extracellular matrix deposition. (**e**) Enhanced type III collagen expression 1 week following injury (green) as visualized by immunocytochemistry. VE-cadherin and DAPI denote endothelial junctions (red) and nuclei (blue), respectively. Scale bar: 60 μm (**f**) Immunocytochemistry for Lamininß1 expression 1 week following injury (green). VE-cadherin and ERG denote endothelial junctions (red) and nuclei (blue), respectively. Scale bar: 60 μm.

Differential gene expression of aortas 1 week following injury identified 526 DEGs, 400 of which were upregulated and 126 of which were downregulated. Gene ontology enrichment of this gene cohort highlighted 238 distinct biological processes (*p* < 0.05, Supplementary Data S4). The 10 most significant GOs are shown in Fig. 6a and included 199 of the 526 DEGs, which were further filtered by read count and p-value to select the top 50 DEGs. Cell adhesion, positive cell migration, and immune system process were the top three GOs represented by the top 50 genes with 16, 12 and 11 genes, respectively.

Cell adhesion genes exhibiting a greater than 2-fold increase included *Mmp14*, *Col18a1*, *Cd44*, *Itgb2*, *Lamb1*, *Col5a1*, *Coro1a*, *Itgb3*, *Mcam* and *Nid1*. Genes belonging to the positive regulation of cell migration GO with greater than 2-fold increases included *Csfr1*, *Myo1f*, *Col1a1*, *Mmp14*, *Col18a1*, *Igf1*, *Lamb1*, *Itgb3*, *Mmp2*, *Mcam* and *Dab2* (Fig. 6b). All of these genes, with the exception of *Dab2*, showed protein-protein interaction scores greater than 0.9 (Fig. 6d, Supplementary Data S4). *Col18a1*, *Lamb1*, *Col5a1*, *Col1a1*, *Lamb2*, *Mmp14*, *Mmp2*, *Itgb3*, *Nid1*, *Igf1*, and *Cd44* formed a tight cluster within this network, stressing the importance of the establishment of the basement membrane and reorganization of endothelial cells as they acclimate following wound closure. Nevertheless, some of the top genes that are differentially expressed at 1 week included those that belong to the immune system process, emphasizing the significant contribution of immune cells at this time point. The cohort of genes within the immune system process that had the largest fold increase were *Lcn2*, *Ltf*, *S100a8*, *Csf1r*, *H2-Ab1*, *H2-Eb1*, *Cd74*, *H2-Aa*, and *Unc93b1* compared to the non-injured aorta (Fig. 6b). With the exception of *H2-Ab1*, *H2-Eb1*, *Cd74*, and *H2-Aa*, genes involved in production of MHC Class II subunits, the remaining genes encoded for proteins with loose clustering via protein-protein interactions and low confidence scores less than 0.75 (Supplementary Data S4). Interestingly, *Itgb2*, *Coro1a*, and *Myo1f*, genes involved in the function and regulation of inflammatory cells, were identified in the analysis of the cell adhesion and positive cell migration ontological processes, suggesting the presence of immune cell binding upon wound closure as endothelial cells reorganize. Protein validation was performed for several of these transcripts, including type III collagen and laminin beta1 (Fig. 6e,f).

Of note, while the transcriptome at this time point predominantly highlights the relevance of cell adhesion, migration and inflammation, cell cycle remained among the top 10 most significant ontological processes. *Mki67* exhibited the largest fold increase of 2.9 with average read counts increasing from 116 ± 19.6 in non-injured aortas to 959 ± 42.6 at 1 week following injury (Fig. 6b and c, Supplementary Data S1). Immunohistochemistry at this time point demonstrated complete wound closure with small foci of EdU-positive cells within aggregates of disorganized endothelial cells (Fig. 2f and f’), suggesting continued endothelial cell turnover despite the presence of a fully repaired endothelial cell monolayer. Furthermore, these foci of endothelial cell proliferation exhibited adherent CD45-positive cells (Suppl. Fig. S3g).

### Regeneration ceases with extracellular matrix remodeling and vessel maturation

**Figure 7:**
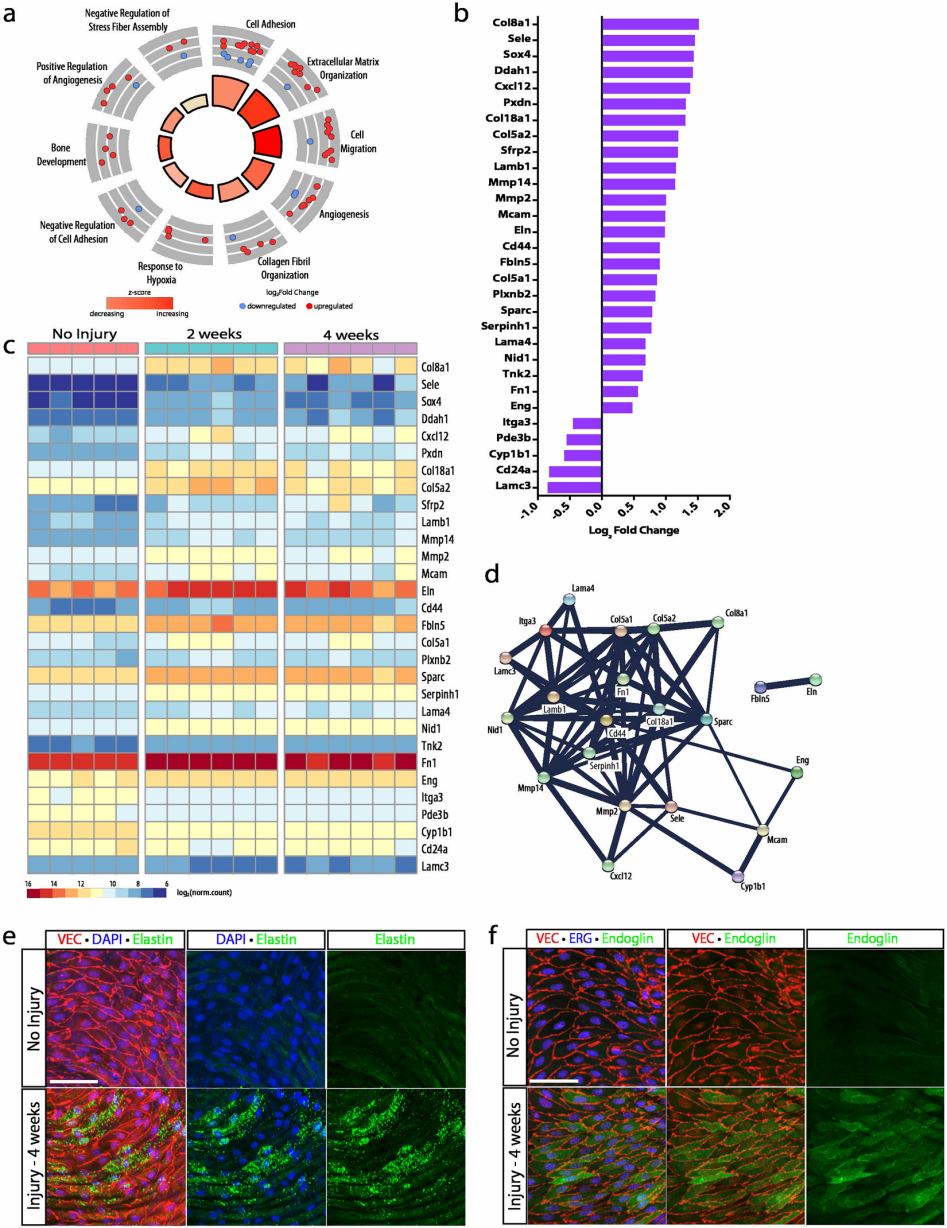
The final stage is marked by elevations in extracellular matrix transcripts. (**a**) Gene ontology enrichment identified cell adhesion and extracellular matrix organization as the top two biological processes at 2 and 4 weeks following injury. (**b**) The top 50 differentially expressed genes (identified by lowest p-value) organized by fold change. (**c**) Heat map showing differences in normalized counts of the top 50 differentially expressed genes between no injury (n = 5), 2 weeks (n = 6), and 4 weeks (n = 6). (**d**) Protein-protein interaction network of top 50 differentially expressed genes via STRING analysis showing clustering of extracellular matrix proteins. (**e**) Immunohistochemistry demonstrates elastin expression 2 weeks following injury (green). VE-cadherin and DAPI reveal endothelial junctions (red) and nuclei (blue), respectively. Scale bar: 60 μm (**f**) Enhanced endoglin expression 4 weeks following injury (green) detected by immunohistochemistry. VE-cadherin and ERG indicate endothelial junctions (red) and nuclei (blue), respectively. Scale bar: 60 μm.

Differential gene expression of aortas at 2 and 4 weeks following injury identified 131 DEGs, of which 83 were upregulated and 48 downregulated. Gene ontology enrichment of this cohort of DEGs was associated with 39 biological processes (*p < *0.05, Supplementary Data S5). The top 10 GOs were identified after filtering for redundant genes and included 42 distinct genes (Fig. 7a). Among these genes, the three activities with the most number of genes were cell adhesion, extracellular matrix organization and angiogenesis, with 10, 8, and 8 genes, respectively. Cell adhesion genes exhibiting a greater than 1.5-fold increase in gene expression included *Col8a1*, *Sele*, *Col18a1*, *Mcam*, *Cd44*, and *Col5a1*. Similarly, genes with a 1.5-fold increase in gene expression within the extracellular matrix organization process include *Pxdn*, *Col18a1*, *Lamb1*, *Eln*, *Fbln5*, *Lama4*, and *Nid1*. Finally, angiogenesis genes exhibiting the same increase in gene expression include *Col8a1*, *Col18a1*, *Mmp14*, *Mmp2*, and *Mcam* (Fig. 7b and c). *Col18a1*, *Col5a1*, *Col8a1*, *Mmp14*, *Mmp2*, *Sele*, *Cd44*, *Nid1*, *Eln*, and *Fbln5* encode for proteins that form interactions with strong confidence scores greater than 0.9 (Fig. 7d, Supplementary Data S5). These protein interactions suggest ongoing cell matrix and basement membrane remodeling leading to vessel maturation. Nevertheless, the low number of DEGs and the low fold change difference in gene expression of this stage compared to other stages suggest that the brunt of vascular regeneration has ceased and only small adjustments in matrix remodeling were still occurring. Unlike other stages, however, the final two-time points were principally focused on remodeling of cell matrix and basement membrane without the contribution of other cellular processes. Immunocytochemistry validated robust expression of elastin and endoglin during this stage (Fig. 7e,f).

### Validation and analysis reveals four stages in vascular regeneration and repair

**Figure 8:**
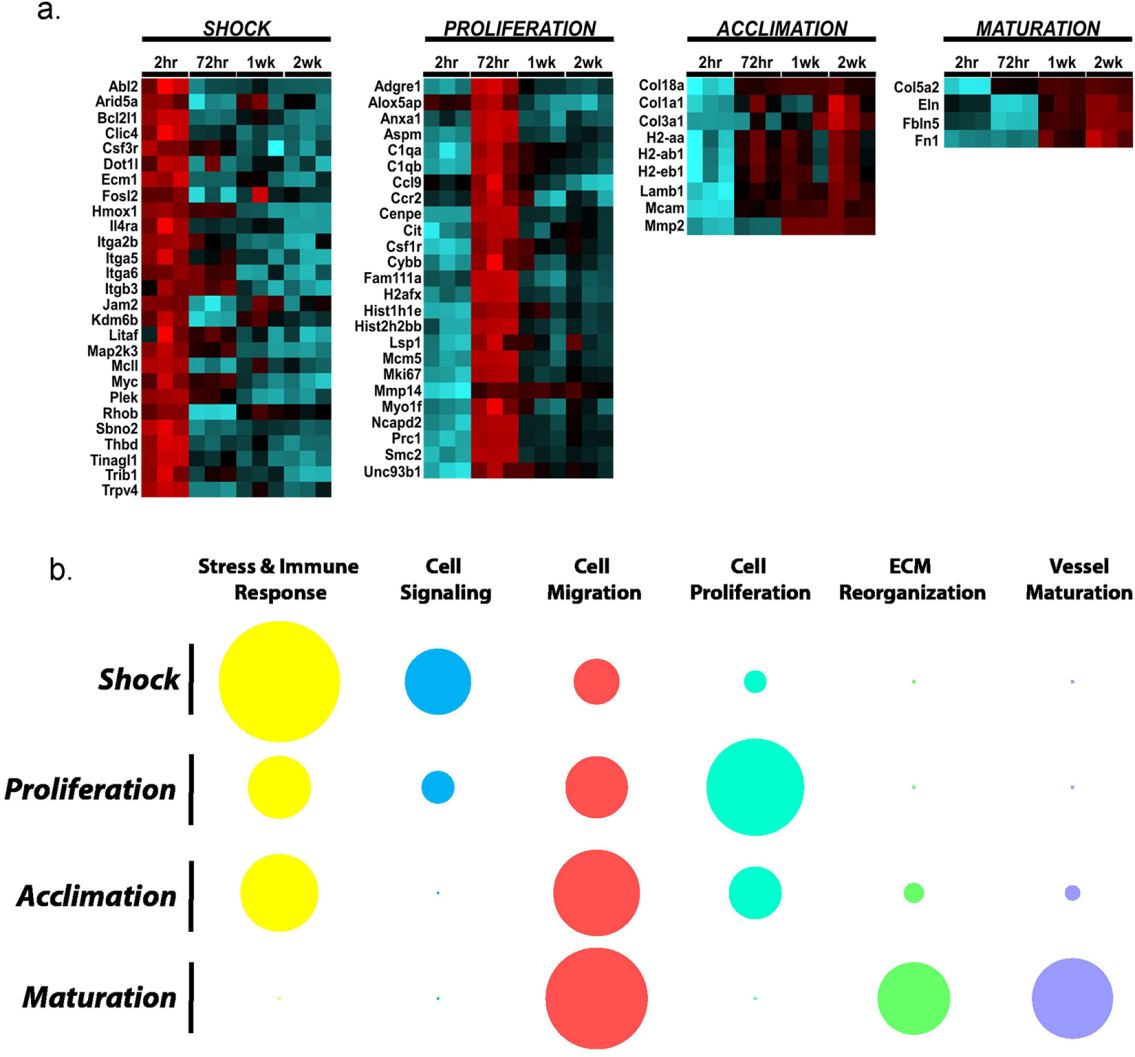
Vascular regeneration can be divided into four distinct transcriptional stages defined by unique changes in cellular processes. (**a**) Differential expression heatmap of a group of genes identified in the RNAseq and now validated using NanoString in post injury intima-enriched aortic samples across multiple time points during wound regeneration (n = 17 [uninjured], n = 30 [2 hr post-injury], n = 17 [72 hr post-injury], n = 13 [1wk post-injury], n = 20 [2wk post-injury]). For each time point, the number of animals were divided into three independent pools for statistical analysis per time point. Statistics included a two-tailed t-test on the log-transformed normalized data assuming unequal variance. Red indicates upregulation in comparison to uninjured intima-enriched aortic samples; blue indicates downregulation. (**b**) Schematic representation of the contribution of stress and immune response, cell signaling, cell migration, cell proliferation, extracellular matrix reorganization and vessel maturation to each transcriptional stage. Cellular processes were categorized based on the top ten GOs identified in each stage. The larger outer circle represented the percentage of all statistically significant DEGs within that category and the darker inner circle represented the proportion of genes within the defined process that are highly expression (base mean > 200 counts). The sum of the inner and outer circle is the percentage of that specific category’s contribution to the total DEG in the top 10 GO categories. The summation of all circles across each row represented all the DEGs in the top 10 categories.

To further confirm the findings from RNAseq, we also performed validation using multiplexed gene expression analysis (NanoString) (Fig. 8a and Supplementary Data S6). The approach enabled the concurrent evaluation of 169 genes identified in the RNAseq and 14 control genes in a single reaction with high sensitivity. The technology has been used recently in several publications with reproducibility and high linearity across a wide range of expression levels^32–34^. Information from these evaluations on a total of 93 cohort of animals supported the RNAseq data and further supported the stages in the process of vascular regeneration and repair (Fig. 8b).

## Discussion

Transcriptional profiling is a powerful tool to understand regulatory interactions and molecular requirements associated with complex biological processes. Here we sought to identify the molecular changes associated with vascular regeneration following mechanical injury *in vivo*. Although previous studies have evaluated transcriptome changes in injured vessels in response to catheter deployment, carotid ligation and atherosclerosis, these evaluations included the entire vessel, largely diluting transcriptional alterations experienced by endothelial cells^35–37^. Using principal component analysis of transcriptomes at defined time points, we showed clustering of samples that departed from the non-injured aorta immediately following injury and then slowly shifted back toward the non-injured aorta (homeostatic point) with time (Fig. 3a). These unique clusters and shifting transcriptional signatures correlated with endothelial proliferation driving wound closure and with subsequent reorganization and remodeling as the events that immediately preceded return to homeostasis. While some of the findings could be anticipated from our understanding of epithelial wound repair, several surprising aspects were also noted. First, the healthy endothelium adjacent to the injury reacted quickly (as fast as 2 hours) to a breach in continuity, likely sensed by tensional forces at cell-cell junctions. Second, there was a rapid, almost synchronically abrupt entry into cell cycle that was not immediately suppressed when endothelial continuity was regained. Third, the signatures noted during repair were distinct from those found during the angiogenic cascade and fourth, active remodeling of the extracellular matrix was the last needed step to regain endothelial vasoregulatory compliance.

Data mining revealed that endothelial repair is a step-wise process that follows four major stages: shock, proliferation, acclimation and maturation (Fig. 8). The first stage, or shock, is characterized by a significant increase in transcripts encoding for acute stressor proteins and integrins as quickly as 2 hours post-injury. Acute stressor transcripts identified included *Fos*, *Fosb*, *Fosl1*, *Fosl2*, *Junb*, and *Jund*, all of which encode for proteins that form a dimeric complex, AP-1 (activator protein-1), and act as transcriptional regulators involved in transduction of signals for proliferation, survival, differentiation and transformation of cells^38^. AP-1 has been found to play a role in mechanical stretch of human osteoblasts and has also been implicated as a mediator between transcriptional regulation and mechanotransduction of shear stress and cyclic strain in aortic endothelial cells^39–42^. Thus, it is likely that in a functional artery AP-1 acts as a central hub for information between junctional tension and proliferative responses. Junctional proteins, particularly but not exclusively VE-cadherin, act as tension sensors and transduce environmental stimuli, such as fluid shear stress, to the cell via cytoskeletal rearrangements^43^. The loss of cell-cell contact following denudation injury, resulting in decreased intensity of VE-cadherin staining along the leading edge of endothelial cells, likely results in changes in mechanical tension and destabilizes cytoskeletal arrangements within the leading edge of cells following injury. Interestingly, both c-Fos and JunB have been implicated in transduction of environmental physical cues in epidermal cells, with upregulation of *Junb* expression following actin depolymerization^44^. As such, loss of tension and junctional integrity along the leading edge of cells may be the trigger that induces expression of these acute stressor transcripts.

In addition to loss of junctional integrity, endothelial denudation injury impacts the integrity of the basement membrane along the wound edge, affecting integrins and focal adhesions. At 2 hours following injury, we found a pronounced transcriptional increase in *Itga6*, *Itga5* and *Itgb3*. Interestingly, there appears to be an emerging link between AP-1 activity, integrin subunit expression and tension during cell migration, suggesting a potential relationship between AP-1 and focal adhesion remodeling. Matrix stiffness was found to upregulate expression of α_6_ integrin by activation of AP-1 in lung myofibroblasts^45^. Yet, contrary to our finding of increased β_3_ integrin and AP-1 transcripts, *in vitro* studies in HUVECs demonstrated repression of α_v_ and β_3_ integrin transcription by dimerization of FOSL1 and JunD in the setting of increased cell adhesion and decreased cell motility^46^.

Following cell shock, endothelial cells were found to quickly enter the cell cycle and proliferate to close the area of denudation. Interestingly, proliferation was isolated to zones along the wound margin rather than be diffusely spread throughout the aorta, suggesting these cells are experiencing a unique stimulus driving cell cycle entry. As previously eluded to, tension may serve as this stimulus acting through junctional proteins. Furthermore, loss of endothelial contact inhibition promotes translocation of β-catenin to the nucleus driving transcription of cell-cycle genes *Myc*, cyclins D1/E2 and VEGF^47,48^. In fact, β-catenin has been shown to regulate transcriptional activation of *Smc2*, a DNA-binding ATPase that is essential for maintenance of chromosomal integrity during cell division, and a gene that was found to be significantly upregulated in our differential expression analysis and validated by evaluation of protein expression by immunohistochemistry^49,50^. *Smc2* was found to interact with several other transcripts involved in mitosis and cell division, including *Ncapd2*, a non-SMC subunit of condensin I, as well as *Mcm5*, *Incenp*, *Cenpe*, *Aspm*, *Prc1*, and *Kif11*. These transcripts, in addition to those involving nucleosome assembly, such as *H2afx*, *Hist1h1*, *Hist1h1a*, *Hist1h1b*, *Hist1h1d*, and *Hist2h2bb*, were identified as the most significant DEGs by p-value and with fold changes ranging from 4-fold to 64-fold increases in gene expression. This signature illustrates a remarkably robust program that drives the cell cycle machinery, promoting cell division and driving wound closure. Nevertheless, while the top 50 DEGs were discussed previously, the proliferative stage exhibited an impressive 2046 total DEGs compromising a host of ontological biological processes, including cell migration, regulation of cell proliferation, positive regulation of transcription, and actin cytoskeletal organization, highlighting the coordination of diverse cellular processes that act concomitantly to repair the denuded area.

The third stage of vascular regeneration is characterized by a robust inflammatory response as well as basement membrane and extracellular matrix remodeling. Along with significant fold increases in *Itgb2* and *Itgam*, suggesting increased inflammatory cell adherence, we observed tight clustering of collagens, laminins, and integrins in the protein interaction network analysis highlighting the importance of basement membrane and ECM deposition during this stage. Gene ontological enrichment also identified the significant contribution of cell cycle genes 1 week following injury, underscoring persistent endothelial cell proliferation among clusters of disorganized endothelium that have lost their polarity. Note that at this time the endothelial wound has been fully closed, and yet proliferation continues. Interestingly, these areas of persistent proliferation and disorganization exhibited preferential and focal adherence of inflammatory cells. The potential homing of inflammatory cells to areas of proliferation might relate to poor junctional integrity as these cells undergo rearrangements in their cell-cell interactions. In fact the adherence of patrolling monocytes to the endothelium was noted in atherosclerotic areas, with the number of monocytes correlating with the level of endothelial cell damage^51^.

As endothelial cells continue to reorganize and polarize in the direction of flow during the final maturation stage (2–4 weeks following injury), we found the predominant processes to include extracellular matrix organization and vessel maturation. These were represented by small fold increases in *Col5a1*, *Col5a2*, *Col8a1*, *Col18a1*, *Nid1*, *and Lamc3*, all of which encode for proteins involved in the maintenance of basement membrane, as well as *Mmp14* and *Mmp2*, highlighting the persistent extracellular matrix remodeling. However, the low number of DEGs at this stage compared to other stages suggests that at this point the regenerated endothelium is functionally recovered and behaving similarly to non-injured endothelium. In fact, further examining genes essential for endothelial cell function, such as *Vegfr1*, *Tie2*, *eNOS*, and *Notch1*, demonstrated a significant decrease in transcript levels at 72 hours and 1 week, both of which have varying but significant levels of proliferation, and a return to non-injured aortic transcript levels by 2 weeks following injury. This was found among all growth factor receptors and vasoregulatory proteins.

It is important to emphasize that our results, although elucidating the biological processes involved in vascular regeneration, reflect transcriptional read outs of the aortic intima and all its components. As such, enrichment was found in both endothelial and inflammatory cells and produced transcriptomes reflective of both cell types. Given their shared hematopoetic lineage, tracing the origin cell of specific transcripts may be problematic. However, the small number of cells involved in regenerating the denudation injury, likely on the order of 50,000 to 100,000, preclude the use of FACS sorting for specific intima-specific cell types. Finally, while our results provide insight into the transcriptional activity of the aortic intima during regeneration, we use protein network analysis to infer potential interactions based on available evidence with established systems for determining confidence of these interactions^52^.

In summary, we defined four transcriptionally unique stages of vascular regeneration following *in situ* injury of large arteries *in vivo* that are functionally distinct from those identified during angiogenesis. We performed deep transcriptomic sequencing and validated the data through immunohistochemistry and an independent Nanostring technology using a large cohort of biological replicates. The combined information allowed us to better define the molecular mechanisms surrounding repair of large arteries. This work provides insight into how regeneration is carried out in the absence of pathological conditions. As such, this study provides baseline information of vascular regeneration and offers the opportunity to significantly build upon our understanding of endothelial functionality in the context of cardiovascular disease.

## Methods

### Animals

Wild type (C57BL/6) mice were obtained from Taconic Biosciences (Hudson, NY). All animals were housed at the University of California, Los Angeles (UCLA) Animal Care Facility and received food and water ad libitum. All animal use protocols, mouse handling, and surgical and experimental procedures were reviewed and approved by the UCLA Institutional Animal Care and Use Committee (IACUC), and these protocols were conducted in accordance with federal regulations as outlined in the “Guide for the Care and Use of Laboratory Animals” prepared by the National Academy of Sciences.

### Denudation Injury

Aortic cross clamping was utilized to produce an arterial denudation injury as described^53^. Briefly, mice were anesthetized with 2.5% isoflurane via nose cone. Laparotomy was performed under a dissecting microscope to expose the infrarenal abdominal aorta. A vascular clamp (Schwartz Micro Serrefines – Sharp Bend Cat# 18052-03, Fine Science Tools, Foster City, CA) was used to cross clamp the aorta for two minutes just proximal to the iliac bifurcation and was sequentially placed proximally to a level just distal to the renal arteries, each time for two minutes. Non-injured mice received a sham surgery involving laparotomy and dissection to the infrarenal abdominal aorta without aortic cross clamping. After injury, mice were housed in a chamber and received carprofen (5 mg/kg, SQ; Norbrook Laboratories, Overland Park, KS) every 24 hours for two days, if appropriate for time point.

### Aorta preparation, EdU staining, immunostaining, and confocal microscopy

Mice were sacrificed after injury at the following time points: 2 hours, 72 hours, 1 week, 2 weeks and 4 weeks. Non-injured mice, which underwent a sham surgery, were also sacrificed. Euthanasia involved isoflurane inhalation and subsequent injection of 5 mg methacholine chloride prepared in phosphate-buffered saline (MP Biochemicals, Santa Ana, CA). Upon confirmation of death by respiratory cessation, mice were perfused with 4% paraformaldehyde in phosphate-buffered saline at a perfusion pressure of 100 mmHg via the left heart for 10 minutes. The intact infrarenal abdominal aorta was subsequently separated from surrounding tissue and transected longitudinally along the dorsal surface. The longitudinally-cut aorta was then incubated in 4% paraformaldehyde in phosphate-buffered saline for at least one hour and pinned on a 35 mm silicone dish with the luminal side up for subsequent *en face* whole-mount immunohistochemistry.

For EdU (5-ethynyl-2′-deoxyuridine) experiments, mice were injected intraperitoneally with 25 mg/kg EdU (Fisher Scientific, Hampton, NH) 2 hours prior to sacrifice and harvest. Following the harvest procedure described above, the remainder of the staining procedure was performed using the Click-iT® Plus EdU Alexa Fluor^®^ 647 Imaging Kit (ThermoFisher Scientific, Waltham, MA) per manufacturer’s protocol. Aortas were subsequently blocked in 1X Hank’s Balanced Salt Solution (HBSS), 3% normal donkey serum, 0.3% Triton-X 100 (Sigma Aldrich, St, Louis, MO), and 0.05% Tween20 (Sigma Aldrich, St, Louis, MO) for one hour. Primary antibodies were applied for at least 60 minutes. Anti-mouse VE-cadherin (sc-6458) was purchased from Santa Cruz Biotechnology. Anti-mouse type III collagen (ab7778), anti-mouse type IV collagen (ab6586), anti-mouse elastin (ab21610), anti-mouse endoglin (ab107595), anti-mouse ERG (ab92513), anti-mouse fibrinogen (ab118533), anti-mouse JunB(ab128878), anti-mouse JunD(ab181615), anti-mouse Ki67 (ab15580), anti-mouse Laminin beta-1 (ab44941) and anti-mouse Smc2 (ab10399) were purchased from Abcam. Anti-mouse CD45 (550539) was purchased from BD). Anti-mouse alpha-smooth muscle actin (F3777) was purchased from Sigma. Anti-mouse CD31 (DIA310) was purchased from Dianova. DAPI (Invitrogen, Carlsbad, CA) was used to stain nuclei when appropriate. A subset of antibodies (CD31, ERG and type IV collagen) were amplified using Tyramide signal amplification kits (T20934 and T20932) purchased from ThermoScientific. Secondary antibodies used included Alexa Fluor 568 donkey anti-goat IgG (A11057), Alexa Fluor 488 donkey anti-sheep IgG (A11015), and Alexa Fluor 488 donkey anti-rat IgG (A21208) purchased from ThermoScientific. A confocal microscope (LSM880, Carl Zeiss) equipped with Zeiss Plan-Apochromat 20x/0.8 M27 was used for image acquisition at room temperature. For 3D reconstructions, confocal z-stacks were imported into Imaris software version 8.0.2 (Bitplane). Multi-color 3D visualized was achieved by iso-surface rendering of each channel.

### RNA isolation, quantification, and qualification

Mice were sacrificed after injury at the following time points: 2 hours (n = 5), 72 hours (n = 6), 1 week (n = 6), 2 weeks (n = 6) and 4 weeks (n = 6). Non-injured mice (n = 8), which underwent a sham surgery, were also sacrificed. Thirty minutes prior to sacrifice, mice were injected with 400 I.U. SQ heparin in phosphate-buffered saline (ThermoFisher Scientific, Waltham, MA). Mice were perfused with 10 mL of phosphate-buffered saline via the left ventricle prior to aortic harvest. The intact infrarenal abdominal aorta was subsequently separated from surrounding tissue and coronally transected immediately distal to the renal arteries and proximal to the iliac bifurcation. Three whole non-injured aortic specimens were further dissected and individually homogenized with lysis buffer. The remaining non-injured (n = 5) and injured aortas at the above-mentioned time points were flushed with 100 ul of lysis buffer pre-warmed to 50 °C through the aortic lumen proximally to distally using an 31-gauge insulin syringe. The flushed material was collected at the iliac bifurcation. Total RNA was subsequently extracted using the RNeasy Micro Kit (Qiagen, Germantown, MD). Contamination with genomic DNA was eliminated by incubation with DNase I (Qiagen, Germantown, MD) for 10 minutes at room temperature. NanoDrop^®^ 8000 (ThermoFisher Scientific, Waltham, MA) was used to measure RNA concentration and purity and integrity was evaluated through analysis on the Agilent Bioanalyzer 2100 system (Agilent Technologies, Santa Clara, CA) was used to assess RNA integrity.

### RNA Sequencing

Library preparation was performed using the SMARTer Stranded Total RNASeq Kit Pico Input (Takara Bio USA, Mountain View, CA) according to manufacturer’s instructions. Sequencing was performed on a HiSeq. 3000 instrument (Illumina, San Diego, CA) using parameters optimized for 50 bp single-end reads at a depth of 20 million reads/sample. Raw sequencing reads were assessed for quality using FastQC^54^, then mapped to mouse strain C57BL6/J genome assembly GRC38.p4^55^ using the HISAT2^56^ algorithm running on the Galaxy platform^57^ with default settings. ENSEMBLE annotation release 84^55^ was used to identify genes, transcripts, protein-coding transcripts, exons, and rRNA for feature counting. Feature counts corrected for genomic DNA contamination were obtained using SeqMonk^58,59^; read counts for genes were determined as counts mapped to the union of all exons for all transcript isoforms of a given gene.

### Analysis of differential expression, gene ontology enrichment and STRING network analysis

Differential expression analysis and generation of the corresponding data plots were performed using the DESeq. 2^60^ package on the Bioconductor platform^61^ using default settings, excepting that the alpha (FDR) threshold was set to 0.01 and a fold change threshold minimum of 2 was imposed. The Bonferonni correction for multiple comparisons, defined by *p* < 0.05 /*m*, where *m* is the number of genes sequenced by RNAseq, was set at 2.6898 × 10^−6^ for judging statistically significant differences in gene expression.

Gene ontology (GO) enrichment analysis was carried out using Database for Annotation, Visualization, and Integrated Discovery (DAVID) 6.8 beta (2016 knowledgebase)^62^ to identify functional classes of genes by biological processes. Biological processes GO terms were queried and significant terms were identified as those with p-values ≤ 0.05. The top ten biological processes for each time point were chosen after removing those processes with greater than 75% redundancy of listed genes when compared to a more statistically significant biological process. The R package GOplot 1.0.2 was used to generate data plots with GO enrichment using default settings^63^. Differentially expressed genes identified within the top ten most significant biological processes were subsequently filtered by p-value and base mean greater than 200, choosing the top 50 genes with lowest p-value and average read count greater than 200. Functional protein association networks of these differentially expressed genes were developed using STRING v10.0 and strength of protein association was determined by confidence score^52^.

### NanoString validation and data analysis

Validation of the data obtained from RNAseq was performed using multiplexed gene expression analysis (NanoString nCounter gene expression assays) using custom-designed gene panel that included 169 genes selected from the RNAseq cohort and 14 control genes. Total RNA was isolated from aortas at the following time points: 2 hrs, 72 hrs, 1week and 2 weeks post-injury to obtain 120 ng total into 8ul. A range of 4–12 animals were pooled to generate one replicate. As three replicates were used, each time point represented a cohort of 13 (72 hrs, 1 w, 2 w) to 30 (2 hrs) animals supporting this analysis with a very robust number of biological replicates. Digital RNA counting and data analyses were performed by NanoString Technologies using nSolver^R^ analysis software. Raw RNA counting data were normalized to the mean of the positive control probes to each assay and to the geometric mean of the 14 housekeeping genes.

### Data availability

All data generated or analyzed during this study are included in this published article (and its Supplementary Information files).

## Electronic supplementary material

Supplementary Figures

S1

S2

S3

S4

S5

S6

## References

[1] Carmeliet P (2005). Angiogenesis in life, disease and medicine. Nature.

[2] Carmeliet P, Jain RK (2011). Molecular mechanisms and clinical applications of angiogenesis. Nature.

[3] Semenza GL (2007). Vasculogenesis, angiogenesis, and arteriogenesis: mechanisms of blood vessel formation and remodeling. Journal of cellular biochemistry.

[4] Phng LK, Gerhardt H (2009). Angiogenesis: a team effort coordinated by notch. Developmental cell.

[5] Trani M, Dejana E (2015). New insights in the control of vascular permeability: vascular endothelial-cadherin and other players. Current opinion in hematology.

[6] Jakobsson L (2010). Endothelial cells dynamically compete for the tip cell position during angiogenic sprouting. Nat Cell Biol.

[7] Herbert SP, Stainier DY (2011). Molecular control of endothelial cell behaviour during blood vessel morphogenesis. Nature reviews. Molecular cell biology.

[8] Simons M, Gordon E, Claesson-Welsh L (2016). Mechanisms and regulation of endothelial VEGF receptor signalling. Nature reviews. Molecular cell biology.

[9] Iruela-Arispe ML, Davis GE (2009). Cellular and molecular mechanisms of vascular lumen formation. Developmental cell.

[10] Domigan CK, Ziyad S, Iruela-Arispe ML (2015). Canonical and noncanonical vascular endothelial growth factor pathways: new developments in biology and signal transduction. Arteriosclerosis, thrombosis, and vascular biology.

[11] Ghaffari S, Leask RL, Jones EA (2015). Flow dynamics control the location of sprouting and direct elongation during developmental angiogenesis. Development (Cambridge, England).

[12] Galie PA (2014). Fluid shear stress threshold regulates angiogenic sprouting. Proceedings of the National Academy of Sciences of the United States of America.

[13] Ricard N, Simons M (2015). When it is better to regress: dynamics of vascular pruning. PLoS biology.

[14] Steele PM (1985). Balloon angioplasty. Natural history of the pathophysiological response to injury in a pig model. Circulation research.

[15] Chaabane C, Otsuka F, Virmani R, Bochaton-Piallat ML (2013). Biological responses in stented arteries. Cardiovascular research.

[16] Otsuka F (2012). The importance of the endothelium in atherothrombosis and coronary stenting. Nature reviews. Cardiology.

[17] Wilensky RL (1995). Vascular injury, repair, and restenosis after percutaneous transluminal angioplasty in the atherosclerotic rabbit. Circulation.

[18] Takeshita S (1994). Increased gene expression after liposome-mediated arterial gene transfer associated with intimal smooth muscle cell proliferation. *In vitro* and *in vivo* findings in a rabbit model of vascular injury. J Clin Invest.

[19] Hassenstein S (1992). Vascular injury and time course of smooth muscle cell proliferation after experimental holmium laser angioplasty. Circulation.

[20] Sakata Y (2004). Transcription factor CHF1/Hey2 regulates neointimal formation *in vivo* and vascular smooth muscle proliferation and migration *in vitro*. Arteriosclerosis, thrombosis, and vascular biology.

[21] Vogt F (2008). Blockade of angio-associated migratory cell protein inhibits smooth muscle cell migration and neointima formation in accelerated atherosclerosis. Journal of the American College of Cardiology.

[22] Majesky MW, Giachelli CM, Reidy MA, Schwartz SM (1992). Rat carotid neointimal smooth muscle cells reexpress a developmentally regulated mRNA phenotype during repair of arterial injury. Circulation research.

[23] Farb A, Shroff S, John M, Sweet W, Virmani R (2001). Late arterial responses (6 and 12 months) after (32)P beta-emitting stent placement: sustained intimal suppression with incomplete healing. Circulation.

[24] Cembrowski MS, Wang L, Sugino K, Shields BC, Spruston N (2016). Hipposeq: a comprehensive RNA-seq database of gene expression in hippocampal principal neurons. eLife.

[25] Henry, F. E., Sugino, K., Tozer, A., Branco, T. & Sternson, S. M. Cell type-specific transcriptomics of hypothalamic energy-sensing neuron responses to weight-loss. *eLife* **4**, 10.7554/eLife.09800 (2015). 10.7554/eLife.09800 PMC4595745 26329458

[26] Eisenhoffer GT (2012). Crowding induces live cell extrusion to maintain homeostatic cell numbers in epithelia. Nature.

[27] Miyoshi H, Ajima R, Luo CT, Yamaguchi TP, Stappenbeck TS (2012). Wnt5a potentiates TGF-beta signaling to promote colonic crypt regeneration after tissue injury. Science (New York, N.Y.).

[28] Bosworth A (2017). A comparison of host gene expression signatures associated with infection *in vitro* by the Makona and Ecran (Mayinga) variants of Ebola virus. Sci Rep.

[29] Sober S (2016). RNA sequencing of chorionic villi from recurrent pregnancy loss patients reveals impaired function of basic nuclear and cellular machinery. Sci Rep.

[30] Ni CW (2010). Discovery of novel mechanosensitive genes *in vivo* using mouse carotid artery endothelium exposed to disturbed flow. Blood.

[31] Nam D (2009). Partial carotid ligation is a model of acutely induced disturbed flow, leading to rapid endothelial dysfunction and atherosclerosis. American journal of physiology. Heart and circulatory physiology.

[32] Kulkarni, M. M. Digital multiplexed gene expression analysis using the NanoString nCounter system. *Current protocols in molecular biolog*y Chapter 25, Unit25B.10, 10.1002/0471142727.mb25b10s94 (2011). 10.1002/0471142727.mb25b10s94 21472696

[33] Bentley-Hewitt KL (2016). Comparison of quantitative real-time polymerase chain reaction with NanoString(R) methodology using adipose and liver tissues from rats fed seaweed. N Biotechnol.

[34] Shukla N, Yan IK, Patel T (2018). Multiplexed Detection and Quantitation of Extracellular Vesicle RNA Expression Using NanoString. Methods Mol Biol.

[35] Ji R (2007). MicroRNA expression signature and antisense-mediated depletion reveal an essential role of MicroRNA in vascular neointimal lesion formation. Circulation research.

[36] Fedorov A (2014). Early changes of gene expression profiles in the rat model of arterial injury. J Vasc Interv Radiol.

[37] Herring BP, Hoggatt AM, Griffith SL, McClintick JN, Gallagher PJ (2017). Inflammation and vascular smooth muscle cell dedifferentiation following carotid artery ligation. Physiol Genomics.

[38] Eferl R, Wagner EF (2003). AP-1: a double-edged sword in tumorigenesis. Nature reviews. Cancer.

[39] Chien S, Li S, Shyy YJ (1998). Effects of mechanical forces on signal transduction and gene expression in endothelial cells. Hypertension (Dallas, Tex.: 1979).

[40] Du W, Mills I, Sumpio BE (1995). Cyclic strain causes heterogeneous induction of transcription factors, AP-1, CRE binding protein and NF-kB, in endothelial cells: species and vascular bed diversity. Journal of biomechanics.

[41] Sumpio BE, Wei D, Wei-Jun X (1994). Exposure of Endothelial Cells to Cyclic Strain Induces c-fos, fosB and c-jun But not jun B or jun D and Increases the Transcription Factor AP-1. Endothelium.

[42] Peverali FA, Basdra EK, Papavassiliou AG (2001). Stretch-mediated activation of selective MAPK subtypes and potentiation of AP-1 binding in human osteoblastic cells. Molecular medicine (Cambridge, Mass.).

[43] Conway DE (2013). Fluid shear stress on endothelial cells modulates mechanical tension across VE-cadherin and PECAM-1. Curr Biol.

[44] Connelly JT (2010). Actin and serum response factor transduce physical cues from the microenvironment to regulate epidermal stem cell fate decisions. Nat Cell Biol.

[45] Chen H (2016). Mechanosensing by the alpha6-integrin confers an invasive fibroblast phenotype and mediates lung fibrosis. Nature communications.

[46] Evellin S (2013). FOSL1 controls the assembly of endothelial cells into capillary tubes by direct repression of alphav and beta3 integrin transcription. Molecular and cellular biology.

[47] Wallez Y, Huber P (2008). Endothelial adherens and tight junctions in vascular homeostasis, inflammation and angiogenesis. Biochim Biophys Acta.

[48] Hecht A, Kemler R (2000). Curbing the nuclear activities of beta-catenin. Control over Wnt target gene expression. EMBO reports.

[49] Davalos V (2012). Human SMC2 protein, a core subunit of human condensin complex, is a novel transcriptional target of the WNT signaling pathway and a new therapeutic target. The Journal of biological chemistry.

[50] Hudson DF, Vagnarelli P, Gassmann R, Earnshaw WC (2003). Condensin is required for nonhistone protein assembly and structural integrity of vertebrate mitotic chromosomes. Developmental cell.

[51] Quintar A (2017). Endothelial Protective Monocyte Patrolling in Large Arteries Intensified by Western Diet and Atherosclerosis. Circulation research.

[52] Szklarczyk D (2015). STRINGv10: protein-protein interaction networks, integrated over the tree of life. Nucleic acids research.

[53] Shirali, A. S., McDonald, A. I., Mack, J. J. & Iruela-Arispe, M. L. Reproducible Arterial Denudation Injury by Infrarenal Abdominal Aortic Clamping in a Murine Model. *Journal of visualized experiments: JoVE*, 10.3791/54755 (2016). 10.3791/54755 PMC5226299 27911412

[54] FastQC: a quality control tool for high throughput sequence data (2010).

[55] Yates A (2016). Ensembl 2016. Nucleic acids research.

[56] Kim D, Langmead B, Salzberg SL (2015). HISAT: a fast spliced aligner with low memory requirements. Nature methods.

[57] Afgan E (2016). The Galaxy platform for accessible, reproducible and collaborative biomedical analyses: 2016 update. Nucleic acids research.

[58] SeqMonk: A tool to visualise and analyse high throughput mapped sequence data (2007).

[59] Andrews, S. B., L. RNA-Seq samples can be contaminated with DNA. QC Fail https://sequencing.qcfail.com/articles/rna-seq-samples-can-be-contaminated-with-dna/ (2016).

[60] Love MI, Huber W, Anders S (2014). Moderated estimation of fold change and dispersion for RNA-seq data with DESeq. 2. Genome biology.

[61] Huber W (2015). Orchestrating high-throughput genomic analysis with Bioconductor. Nature methods.

[62] Huang da W, Sherman BT, Lempicki RA (2009). Systematic and integrative analysis of large gene lists using DAVID bioinformatics resources. Nature protocols.

[63] Wencke, W., Sanchez-Cabo, F. & Ricote, M. GOplot: an R package for visually combining expression data with functional analysis. *Bioinformatics***btv300** (2015). 10.1093/bioinformatics/btv300 25964631

