# Supplementary material for: A multi-step transcriptional cascade underlies vascular regeneration in vivo
Source: Sci Rep. 2018 Apr 3;8:5430. doi: 10.1038/s41598-018-23653-3 (PMC5882937; doi:10.1038/s41598-018-23653-3)

Supplementary Information For:

"A multi-step transcriptional cascade underlies vascular regeneration in vivo"

Aditya S. Shirali, Milagros C. Romay, Austin I. McDonald, Trent Su, Michelle E. Steel, M. Luisa Iruela-Arispe

Supplementary Figures:

Supplementary Figure S1: Endothelial denudation injury model (a) Representative denudation injury from three aortas shown *en face*. VE-cadherin was used to visualized endothelial cells (red). Denudation areas are highlighted in white. (b) Average of independent denudation injuries. (c) Longitudinal sections of dorsal aortae that were either uninjured, injured (48hrs) or regenerated (1 month) were evaluated for expression of Collagen IV and CD31. Scale bar: left images 100um, right images 20um. EC= endothelial cells; BM= basement membrane.

Supplementary Figure S2: Generation of intima-enriched RNA samples. (a) Schematic representation of RNA collecting procedure. Lysis buffer was flushed through aorta to collect intima-enriched aortic RNA. (b) Transversal sections of aortae either flushed with vehicle or RNA lysis buffer and evaluated for expression of ERG (endothelial marker) and alpha-smooth muscle actin (smooth muscle marker). (b') Higher magnification of region indicated by the white rectangle in (b). (c) Principal Component Analysis (PCA) demonstrated the similarities between samples isolated from the tunica intima-enriched (blue), flushed aorta – tunica media-enriched (green), or whole aorta (red). (d) Heat map showing Euclidean distances between the samples as calculated from the normalized log change. (e) Heat map showing relative expression of endothelial markers, smooth muscle cell markers, white blood cell-specific markers, platelet markers, and NG-2, a pericyte marker. Note enrichment of intima-enriched aortic samples with endothelial-specific markers and decreased smooth muscle cell markers.

Supplementary Figure S3: Denudation injury induces accumulation of CD45-positive cells within the wound area.
(a-f) Relative abundance of CD45-positive cells (white) at the indicated times post-injury. VE-cadherin was used to visualize endothelial junctions (red). (a')-(f') Higher magnification of boxed areas. Scale bar: 40um. (g) Adherence of CD45 positive cells (white) to foci of EdU-positive cells (green) at 72hours, 1 week, and 2 weeks following injury. VE-cadherin was used to visualize endothelial junctions (red). Scale bar: 40um.

Supplementary Data:

Supplementary Data S1: Mean DESeq2 normalized read counts for all transcripts in non-injured whole aortic samples (n=3), non-injured intima-enriched aortic samples (n=5), injured intima-enriched aortic samples at 2 hours post-injury (n=5), injured intima-enriched aortic samples at 72 hours post-injury (n=6), injured intima-enriched aortic samples at 1 week post-injury (n=6), injured intima-enriched aortic samples at 2 weeks post-injury (n=6), and injured intima-enriched aortic samples at 4 weeks post-injury (n=6).
(a separate .xls file, 3.5 MB)

Supplementary Data S2: Differentially expressed genes (DEGs) between non-injured intima-enriched aortic samples (n=5) and injured intima-enriched aortic samples at 2 hours post-injury (n=5, highlighted genes represent those that are statistically significant). Gene ontology enrichment analysis of biological processes of DEGs. Protein-protein interaction network analysis of top 50 DEGs.
(a separate .xls file, 1.4 MB)

Supplementary Data S3: Differentially expressed genes (DEGs) between non-injured intima-enriched aortic samples (n=5) and injured intima-enriched aortic samples at 72 hours post-injury (n=6, highlighted genes represent those that are statistically significant). Gene ontology enrichment analysis of biological processes of DEGs. Protein-protein interaction network analysis of top 50 DEGs.
(a separate .xls file, 1.4 MB)

Supplementary Data S4: Differentially expressed genes (DEGs) between non-injured intima-enriched aortic samples (n=5) and injured intima-enriched aortic samples at 1 week post-injury (n=6, highlighted genes represent those that are statistically significant). Gene ontology enrichment analysis of biological processes of DEGs. Protein-protein interaction network analysis of top 50 DEGs.
(a separate .xls file, 1.3 MB)

Supplementary Data S5: Differentially expressed genes (DEGs) between non-injured intima-enriched aortic samples (n=5) and injured intima-enriched aortic samples at 2 and 4 weeks post-injury (n=6 for each time point, highlighted genes represent those that are statistically significant). Gene ontology enrichment analysis of biological processes of DEGs. Protein-protein interaction network analysis of top 50 DEGs.
(a separate .xls file, 1.6 MB)

Supplementary Data S6: Summary of results for nanostring validation of selected deferentially expressed genes (DEG) between non-injured intima enriched aortic samples and injured intima-enriched aortic samples across multiple time points (n=17 [uninjured], n=30[2hr], n=17[72hr], n=13[1wk], n=20[2wk], forming three independent pools for statistical analysis per time point). Statistics were performed using a two-tailed t-test on the log-transformed normalized data assuming unequal variance.

Supplementary Figure S1

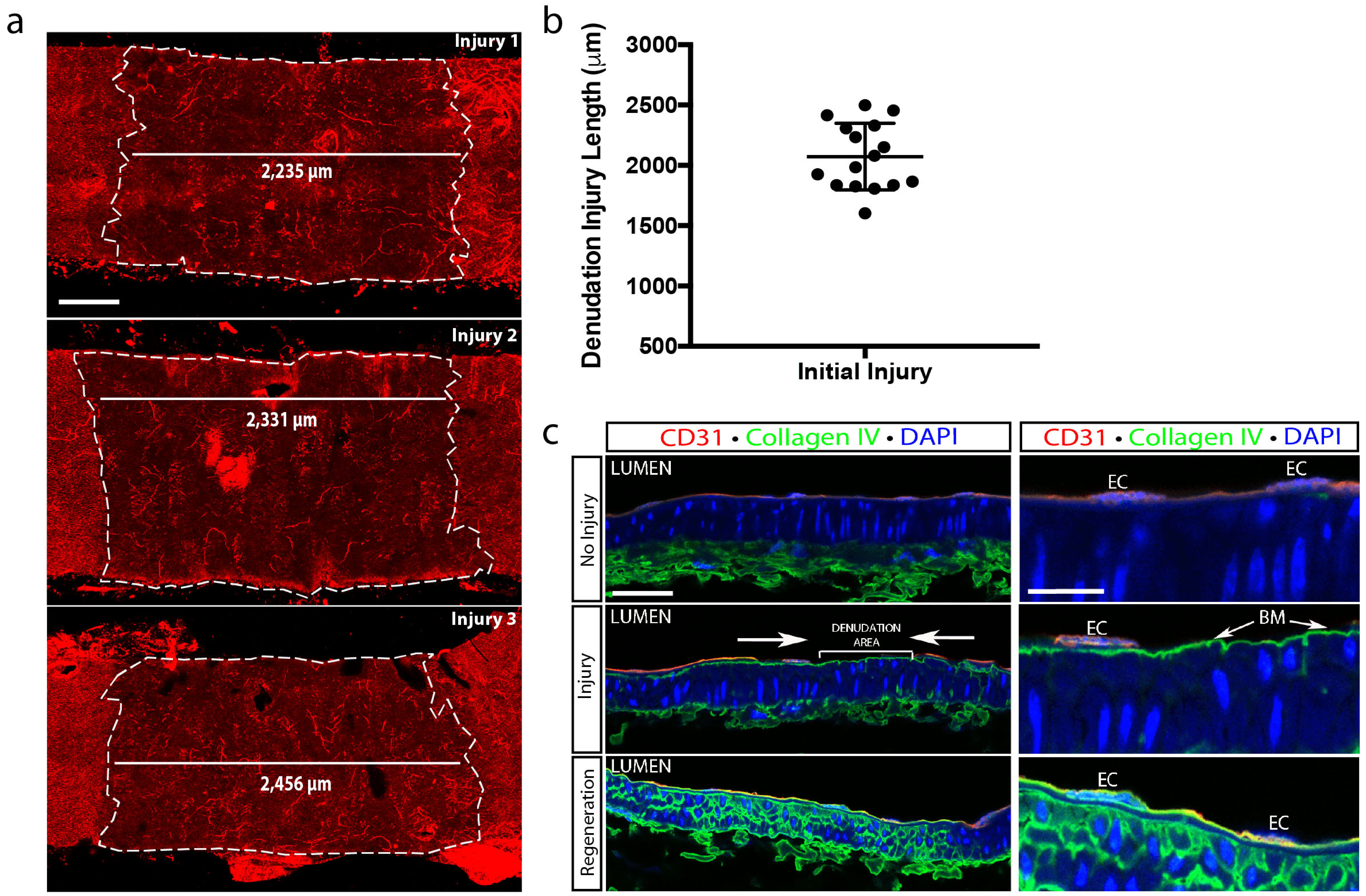

Supplementary Figure S2

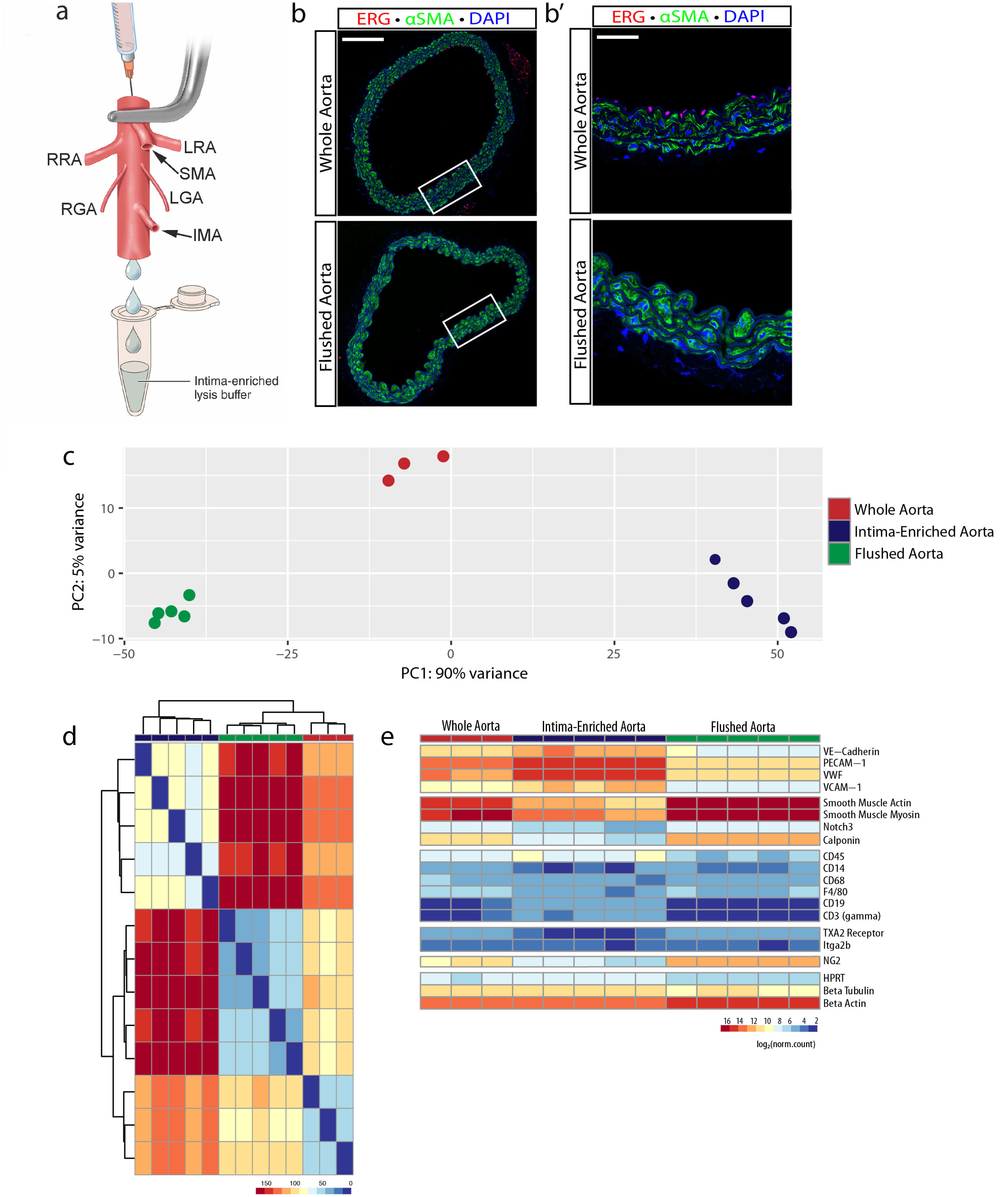

Supplementary Figure S3

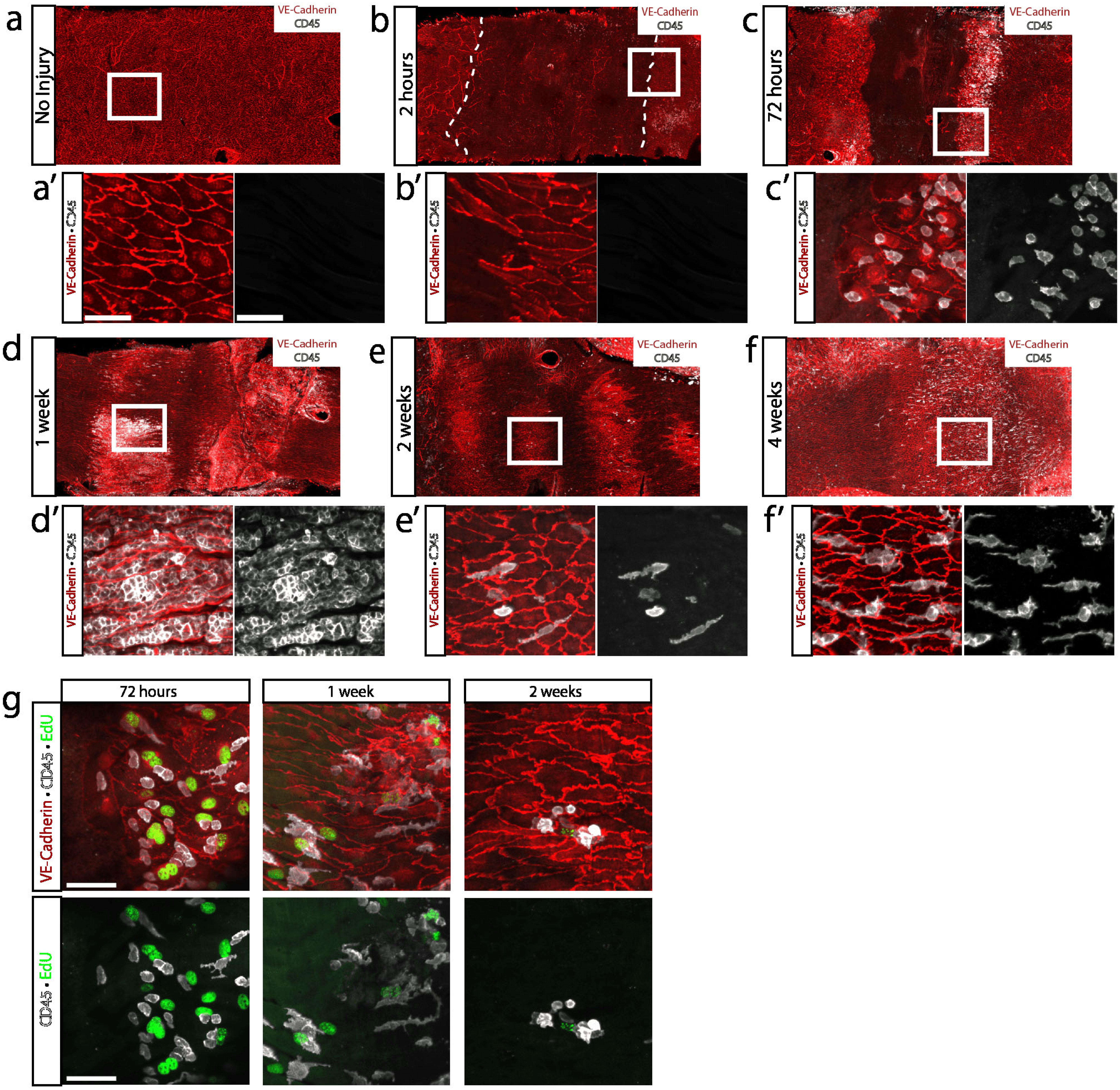

Supplement: Supplementary file 1 — Supplementary Figures [file 41598_2018_23653_MOESM1_ESM.pdf]
